# Leaf Essential Oil Compositions and Enantiomeric Distributions of Monoterpenoids in *Pinus* Species: *Pinus albicaulis*, *Pinus flexilis*, *Pinus lambertiana*, *Pinus monticola*, and *Pinus sabiniana*

**DOI:** 10.3390/molecules30020244

**Published:** 2025-01-09

**Authors:** Alicia Moore, Elizabeth Ankney, Kathy Swor, Ambika Poudel, Prabodh Satyal, William N. Setzer

**Affiliations:** 1 Independent Researcher, 6346 Pentz Rd., Paradise, CA 95969, USA; 2 Independent Researcher, 141 W. 17th St., Lafayette, OR 97127, USA; 3 Independent Researcher, 1432 W. Heartland Dr., Kuna, ID 83634, USA; 4 Aromatic Plant Research Center, 230 N 1200 E, Suite 100, Lehi, UT 84043, USApsatyal@aromaticplant.org (P.S.); 5 Department of Chemistry, University of Alabama in Huntsville, Huntsville, AL 35899, USA

**Keywords:** whitebark pine, limber pine, sugar pine, western white pine, gray pine, gas chromatography, enantioselective, chiral

## Abstract

Members of the *Pinus* genus are well known for their medicinal properties, which can be attributed to their essential oils. In this work, we have examined the leaf essential oils of five understudied *Pinus* species collected from various locations in western North America. The essential oils were obtained by hydrodistillation and analyzed by gas chromatographic methods, including enantioselective gas chromatography. *Pinus albicaulis* was dominated by (+)-δ-3-carene; *Pinus flexilis* was dominated by α-pinene (mostly (+)-α-pinene) and (−)-β-pinene; *Pinus lambertiana* was dominated by (−)-β-pinene; *Pinus monticola* was dominated by (−)-β-pinene, (+)-δ-3-carene, and (−)-α-pinene; and *Pinus sabiniana* was rich in (−)-α-pinene and limonene. While this work adds to our knowledge of *Pinus* essential oils, additional research is needed to more fully appreciate the geographic and altitudinal variations in the volatile compositions of these *Pinus* species.

## 1. Introduction

The genus *Pinus* L. is composed of around 134 species, distributed throughout the northern hemisphere, and introduced to several locations in the southern hemisphere [1]. Members of the *Pinus* genus are valued for their medicinal properties [2,3,4], which can be largely attributed to their essential oils. Common components in pine essential oils include α-pinene, β-pinene, myrcene, δ-3-carene, camphene, limonene, and β-phellandrene, among others [2]. In this study, several unstudied or understudied *Pinus* species from western North America have been obtained and analyzed using gas chromatographic methods.

**Figure 1:**
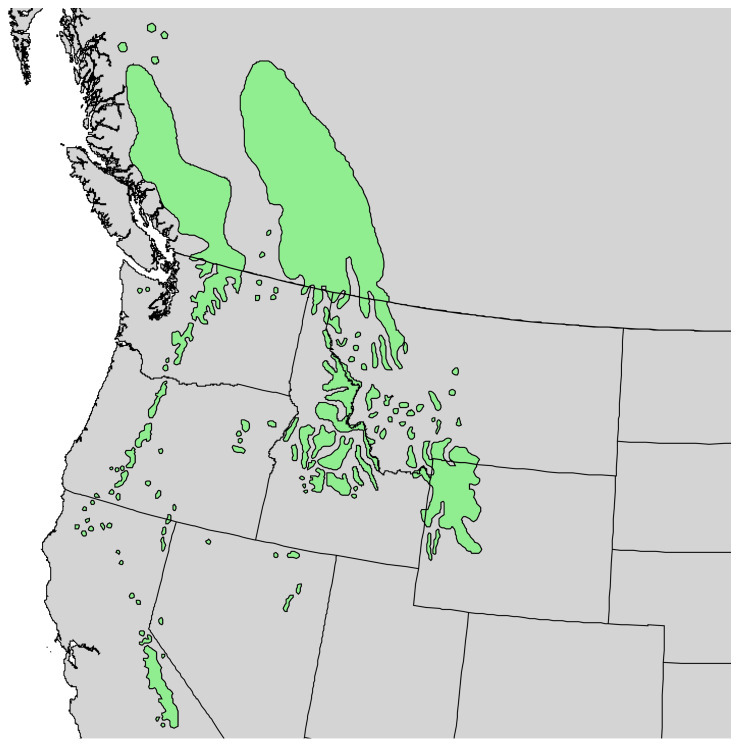
Range of *Pinus albicaulis* Engelm. [11].

**Figure 2:**
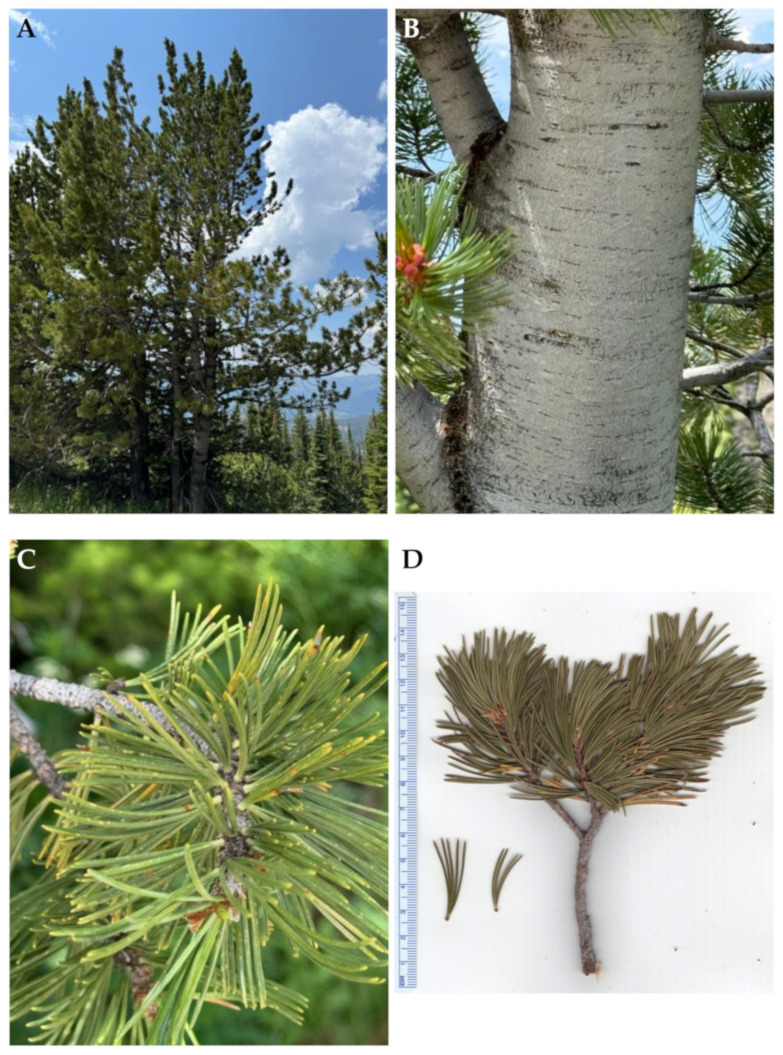
*Pinus albicaulis* Engelm. (**A**): The tree habit. (**B**): Bark. (**C**): Leaves. (**D**): A scan of a twig with leaves. Photographs by K. Swor at the time of collection.

*Pinus albicaulis* Engelm. (whitebark pine) is an evergreen monoecious tree found in Alpine habitats in western North America, including the Cascade, Rocky Mountain, and Sierra Nevada ranges (Figure 1) [5]. The trees grow up to 21 m tall, the bark is gray, and the leaves (needles) are five per fascicle (Figure 2). Whitebark pine is considered to be a keystone species, providing food for birds and mammals, slowing snowmelt runoff, and reducing soil erosion. Whitebark pine has co-evolved with Clark’s nutcracker (*Nucifraga columbiana* (A. Wilson)), which disperse the seeds of the tree [6]. Unfortunately, the population of this tree species has been declining at an alarming rate due to the invasive pathogen *Cronarium ribicola* J.C. Fisch., which causes blister rust, large-scale outbreaks of the mountain pine beetle (*Dendroctonus ponderosae* Hopkins), altered fire regimes from fire exclusion, and more frequent and severe fires due to climate change [7,8]. Whitebark pine deaths have increased from 43% to 54% from 2010 to 2019 [9]. Whitebark pine has recently (2022) been classified by the Endangered Species Act as a “threatened species” [10]. As far as we are aware, there have been no previous reports on the essential oil composition of *P. albicaulis*; identification of the volatile phytochemicals in whitebark pine may be useful in identifying potential bark beetle repellents or antifungal agents.

**Figure 3:**
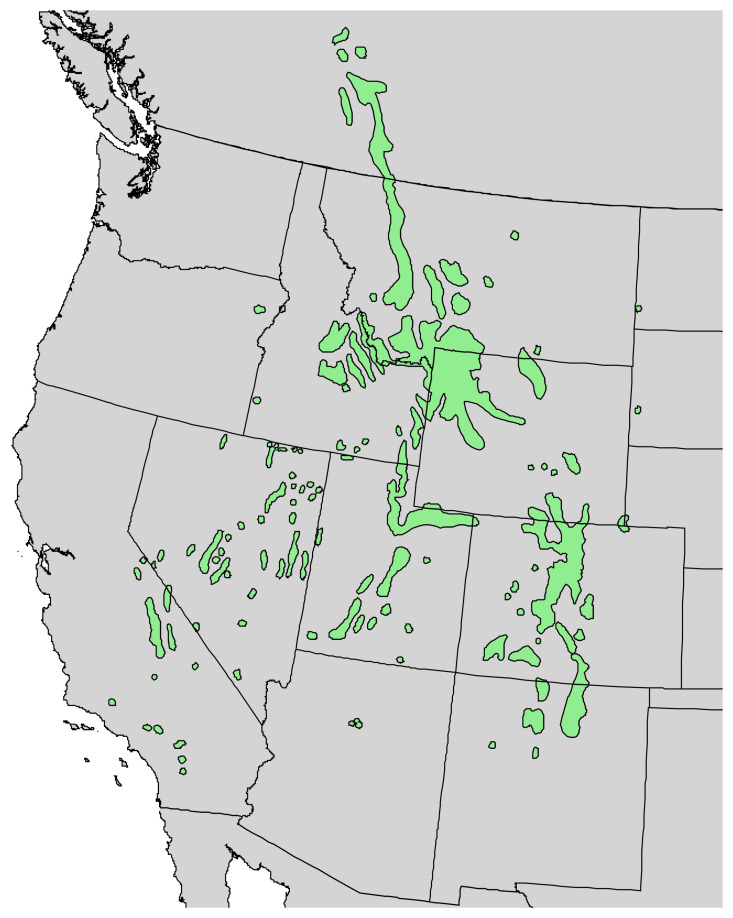
Natural range of *Pinus flexilis* E. James [11].

**Figure 4:**
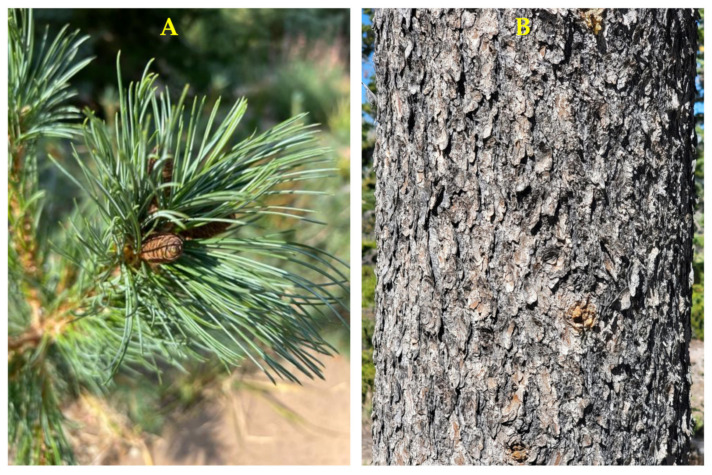
*Pinus flexilis* E. James. (**A**): A branch with leaves (needles). (**B**): Bark. Photographs by K. Swor at the time of collection.

*Pinus flexilis* E. James (limber pine) is a five-needle pine species [9]. The tree is found in the Rocky Mountains of western North America, from southeastern British Columbia to southwestern Alberta, and south through Colorado and New Mexico. Limber pine is also found in the mountains of Utah, Idaho, Nevada, and California [12] (Figure 3). The tree grows up to 26 m tall, with gray bark and five needles per fascicle (Figure 4). The Navajo people used *P. flexilis* as a febrifuge, emetic, and cough medicine [13]. There has been one previous report on the leaf essential oil of *P. flexilis* from Idaho [14].

**Figure 5:**
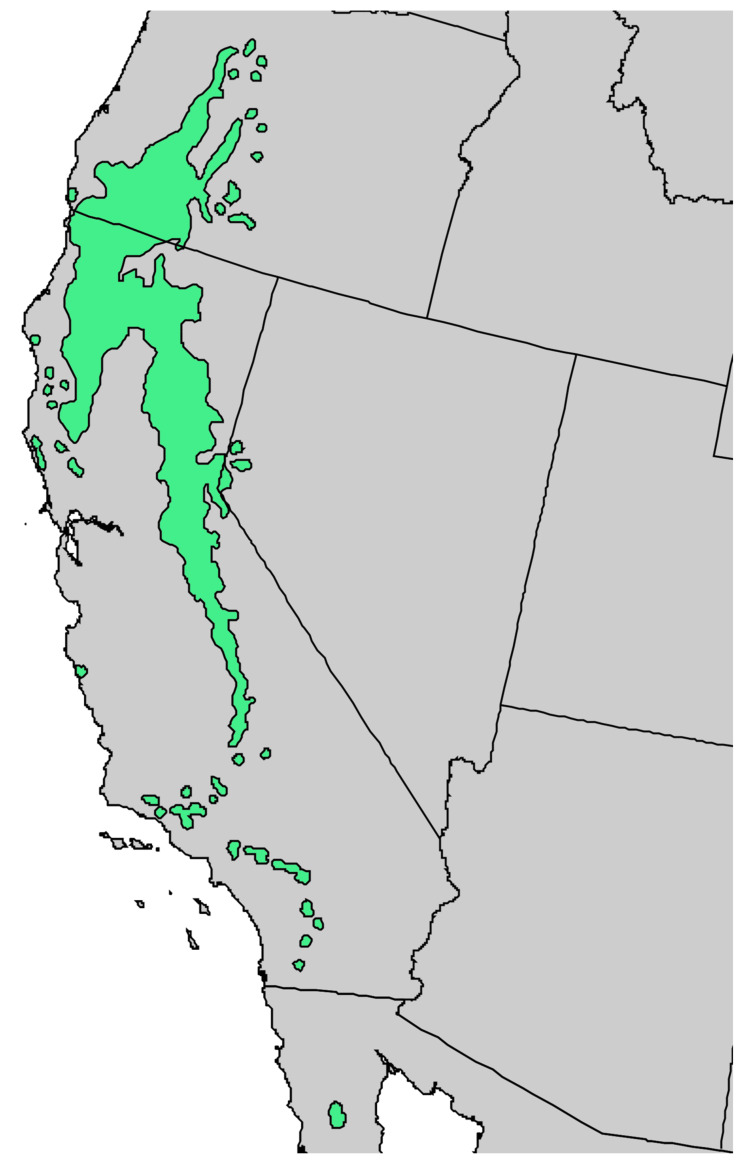
Natural range of *Pinus lambertiana* Douglas [11].

**Figure 6:**
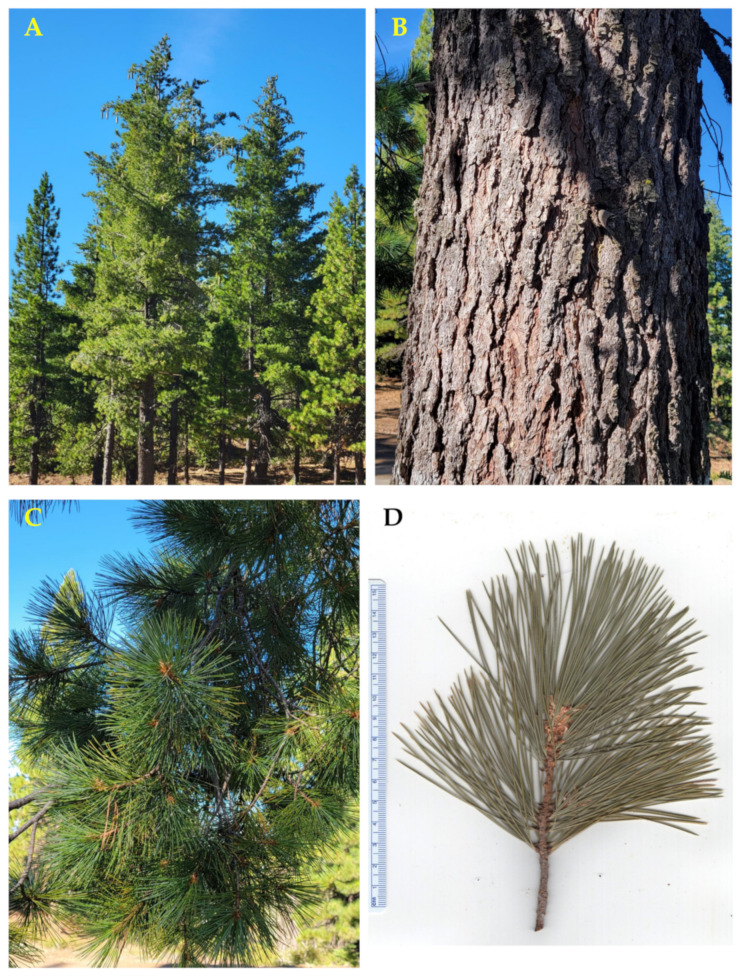
*Pinus lambertiana* Douglas. (**A**): The tree habit. (**B**): Bark. (**C**): Leaves (needles). (**D**): A scan of a twig with leaves. Photographs by A. Moore at the time of collection.

*Pinus lambertiana* Douglas (sugar pine) is found in montane dry to moist forests in western North America from Oregon, south through California, and into Baja California (Figure 5) [15]. These are very large trees, growing up to 75 m tall; the bark is cinnamon- to gray-brown in color and deeply furrowed. There are five leaves (needles) per fascicle; the seed cones are large (25–50 cm), yellow-brown, and resinous (Figure 6) [16]. The Mendocino Native Americans used *P. lambertiana* as a cathartic [17]. As far as we are aware, there have been no previous reports on the essential oil of *P. lambertiana*.

**Figure 7:**
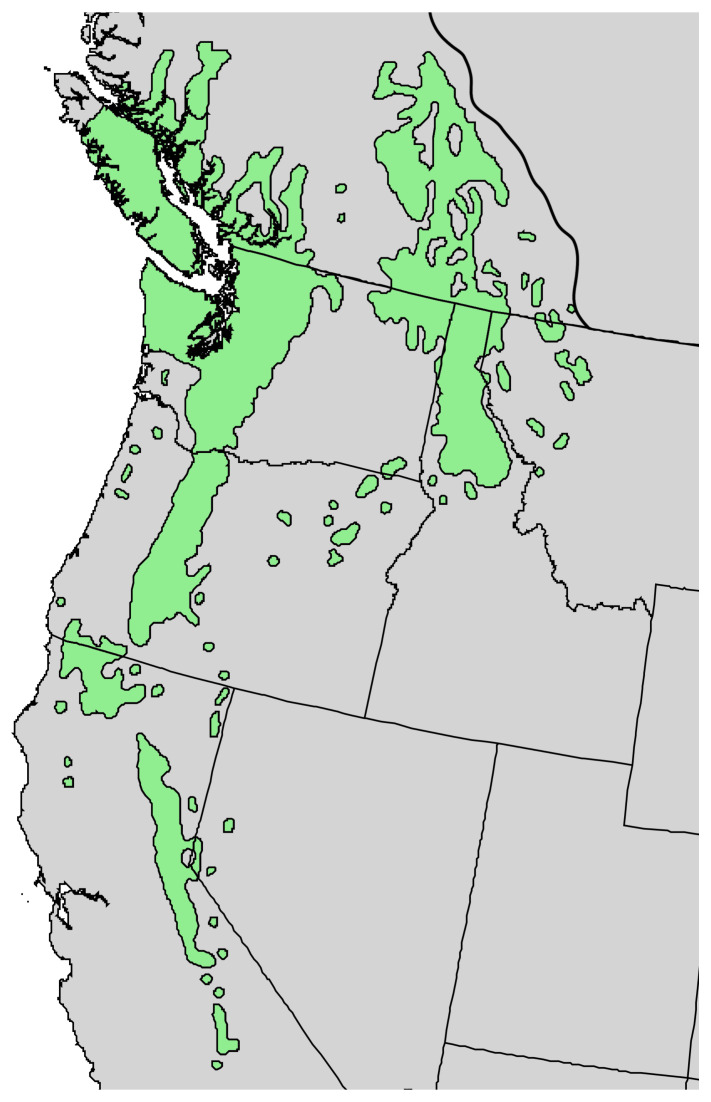
Natural range of *Pinus monticola* Douglas ex. D. Don. [11].

**Figure 8:**
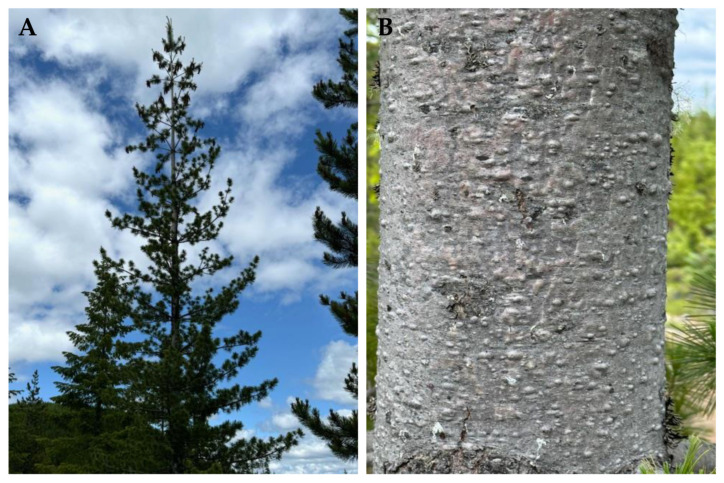

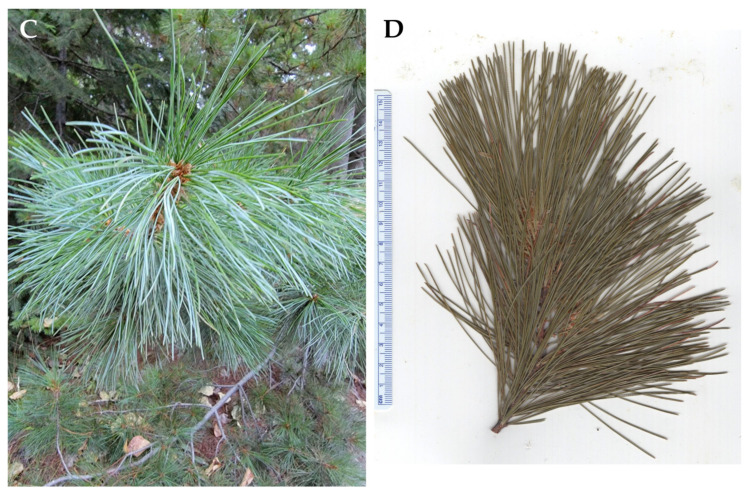
*Pinus monticola* Douglas ex. D. Don. (**A**): The tree habit. (**B**): The bark of a young tree. (**C**): Leaves (needles). (**D**): A scan of leaves. Photographs by K. Swor at the time of collection.

*Pinus monticola* Douglas ex D. Don (western white pine) is a large tree that is found in western North America from British Columbia; south through Washington, Montana, Idaho, Nevada, and Oregon; and into California (Figure 7) [18]. The tree is 30–50 m, up to 70 m tall; the bark is grey and smooth, becoming furrowed into hexagonal scaly plates in large individuals. There are five needles per fascicle; the seed cones are 10–25 cm long, creamy brown to yellowish (Figure 8) [18]. The Mahuna Native Americans of California took the plant internally to treat rheumatism [19]. There has been one previous report on the leaf essential oil of cultivated *P. monticola* from Argentina [20].

**Figure 9:**
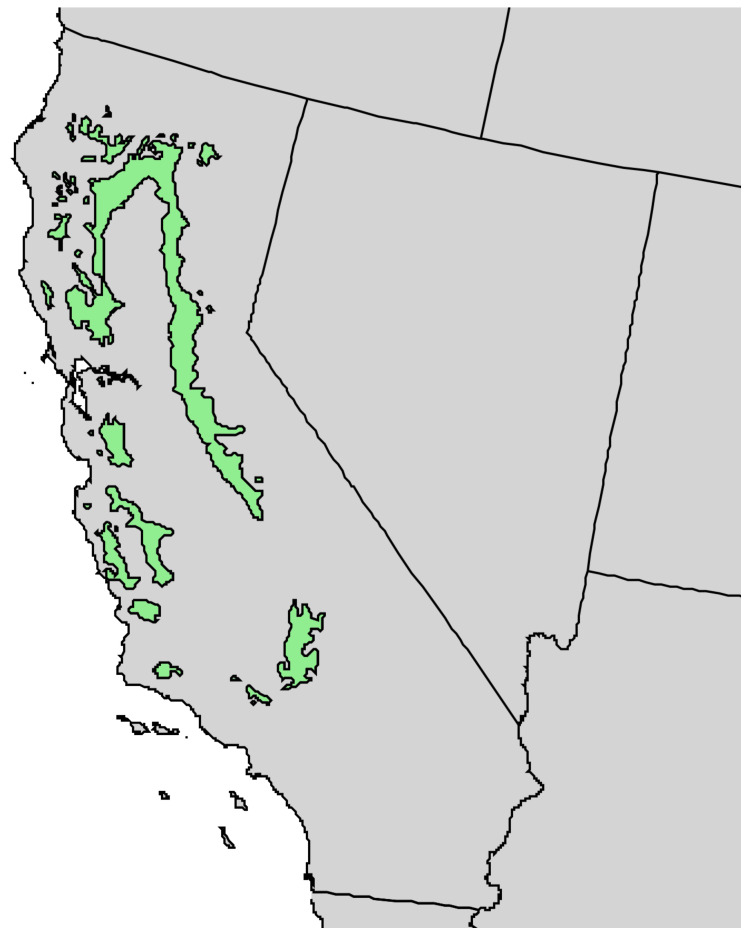
Natural range of *Pinus sabiniana* Douglas in California [11].

**Figure 10:**
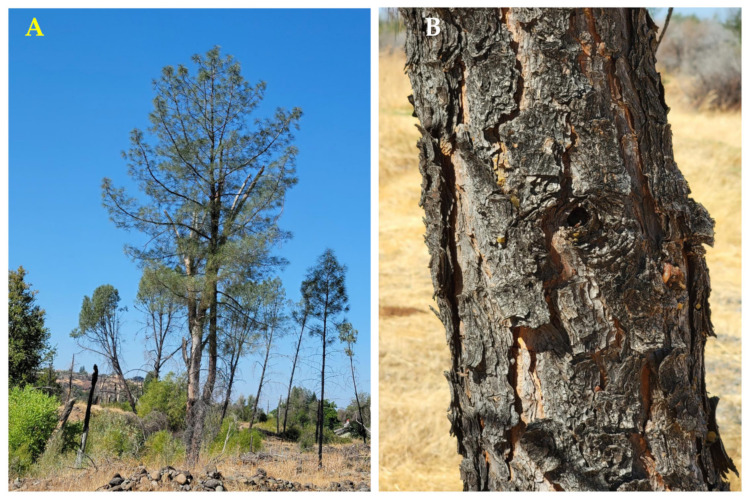

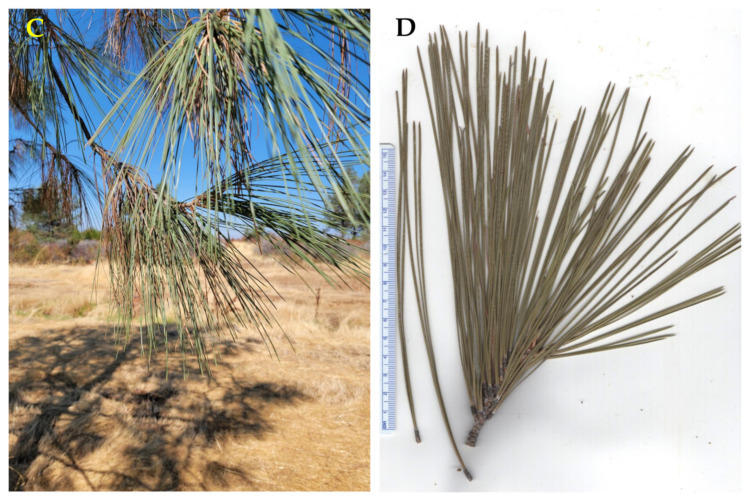
*Pinus sabiniana* Douglas. (**A**): The habit of the tree. (**B**): Bark. (**C**): Leaves (needles). (**D**): A pressed sample of leaves. Photographs by A. Moore at the time of collection.

*Pinus sabiniana* Douglas (gray pine, foothill pine) is endemic to the dry foothills of the coast range and the Sierra Nevada Range in California, essentially encircling the Central Valley (Figure 9) [21]. The trees grow to 25 m tall, the bark is brown to near black and deeply furrowed, there are generally three needles per fascicle (15–32 cm long), and the seed cones are large (15–25 cm) (Figure 10) [22]. The Yuki people of California used the burning twigs and leaves of *P. sabiniana* as a sweat bath for rheumatism and bruises [17]. There has been one previous report on the leaf and wood essential oils of *P. sabiniana* [23].

## 2. Results and Discussion

### 2.1. Pinus albicaulis Engelm

**Figure 11:**
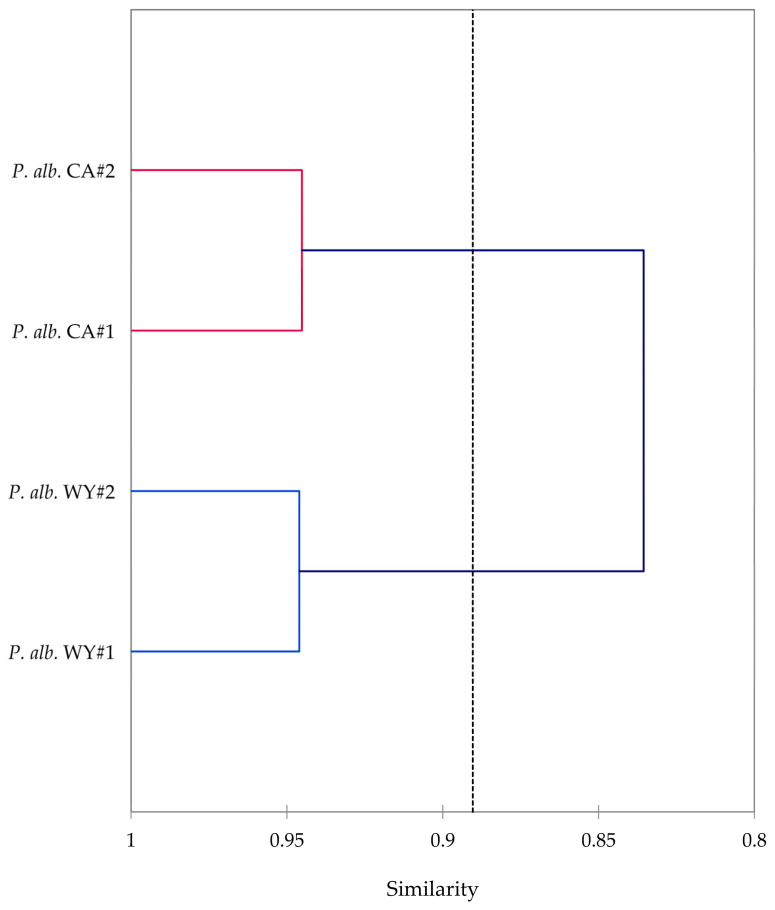
A dendrogram based on an agglomerative hierarchical cluster analysis of the major components (δ-3-carene, α-pinene, β-pinene, limonene, myrcene, β-phellandrene, α-terpinyl acetate, α-cadinol, terpinolene, camphene, α-terpineol, and germacrene D) in the leaf essential oils of *Pinus albicaulis*.

Leaf essential oils of *P. albicaulis* were obtained from sites in Wyoming and California in yields ranging from 2.96% to 3.51%. The essential oil compositions of *P. albicaulis* are presented in Table 1. A total of 106 components were identified in the *P. albicaulis* essential oils, accounting for more than 99% of the compositions. The major components were δ-3-carene (22.0–37.3%), α-pinene (7.8–12.6%), limonene (6.8–9.7%), β-phellandrene (2.0–11.3%), myrcene (3.0–7.1%), α-terpinyl acetate (0.2–14.4%), and terpinolene (3.3–5.1%). The essential oils from the Wyoming trees and the California trees are qualitatively similar and show only minor quantitative differences. That is, agglomerative hierarchical cluster analysis (HCA) shows 84% similarity between the two collection sites (Figure 11). Furthermore, two-sample *t*-test comparisons between the major components are not significantly different (Table 2).

### 2.2. Pinus flexilis E. James

Leaf essential oils were obtained from three individual trees growing in southern Utah in yields of 4.51%, 3.99%, and 5.02%. A total of 86 components were identified in the leaf essential oils of *P. flexilis*, which accounted for 99.6%, 99.9%, and 99.9% of the compositions. The leaf essential oil compositions of *P. flexilis* are listed in Table 3. The essential oils were dominated by β-pinene (11.1–54.8%) and α-pinene (19.8–37.5%), with lesser percentages of limonene (2.7–7.4%), β-phellandrene (1.8–5.1%), α-terpineol (1.5–4.9%), and α-cadinol (1.9–8.3%). The chemical compositions of the samples from southern Utah are qualitatively similar to a *P. flexilis* sample from southwestern Idaho, which showed α-pinene (37.1%) and β-pinene (21.9%) as the major components [14].

### 2.3. Pinus lambertiana Douglas

Leaves of *P. lambertiana* were collected near Butte Meadows, California, and hydrodistilled to give colorless essential oils in yields ranging from 2.01% to 2.26%. The gas chromatographic analysis revealed compositions of 126 total identified components (Table 4). The major components in the essential oils were β-pinene (28.9–46.4%), α-pinene (11.4–20.3%), (*E*)-β caryophyllene (2.5–16.6%), germacrene D (4.4–13.0%), and α-terpineol (2.6–6.9%).

### 2.4. Pinus monticola Douglas ex D. Don

Needles of *P. monticola* were collected from three individual trees located on a lahar slope of Mt. St. Helens, Washington, and three individual trees located near Priest Lake, Idaho. Hydrodistillation gave colorless essential oils ranging from 1.71% to 2.03%. The gas chromatographic analysis led to the identification of a total of 114 components (Table 5). The major leaf oil components were β-pinene (16.7–25.6%), α-pinene (9.8–15.7%), δ-3-carene (8.2–12.9%), limonene (3.7–8.2%), β-phellandrene (3.4–6.2%), myrcene (3.9–4.9%), α-terpineol (2.1–7.8%), α-cadinol (0.7–6.9%), and *trans*-β-elemene (0.7–15.2%). A previous examination of *P. monticola*, cultivated in Valle Chico, Argentina, was found to show β-pinene (22.8%), α-pinene (21.0%), limonene (14.0%), isobornyl formate (5.7%), terpinolene (5.1%), myrcene (4.2%), and δ-3-carene (4.2%) as major components [20].

**Figure 12:**
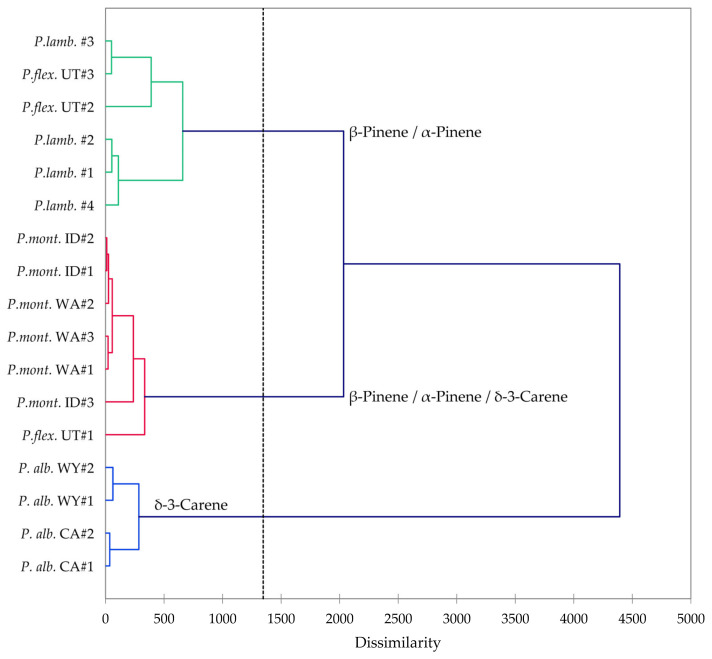
Dendrogram based on hierarchical cluster analysis of leaf essential oil compositions of *Pinus albicaulis*, *Pinus flexilis*, *Pinus lambertiana*, and *Pinus monticola*.

**Figure 13:**
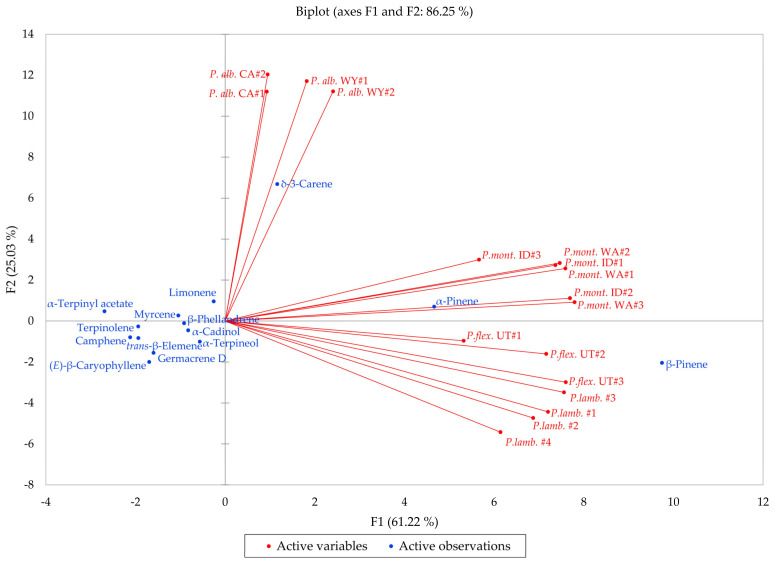
Biplot based on principal component analysis of *Pinus albicaulis*, *Pinus flexilis*, *Pinus lambertiana*, and *Pinus monticola* leaf essential oil compositions.

*Pinus albicaulis*, *P. flexilis*, *P. lambertiana*, and *P, monticola* are all members of the subgenus *Strobus*, section *Quinquefoliae*, subsection *Strobus* (the five-needle white pine group). In order to investigate the similarities and differences in volatile phytochemicals in these species, a hierarchical cluster analysis (HCA) and a principal component analysis (PCA) were carried out. The HCA shows three well-defined clusters based on the essential oil compositions (Figure 12): (a) a cluster dominated by β-pinene and α-pinene and made up of samples of *P. flexilis* and *P. lambertiana*; (b) a cluster dominated by δ-3-carene and made up of *P. albicaulis* samples from northern Wyoming and from northern California; and (c) a cluster defined by relatively large percentages of β-pinene, α-pinene, and δ-3-carene, composed largely by samples of *P. monticola* from Idaho and from Washington. The PCA further delineates the species based on essential oil compositions (Figure 13). The *P. albicaulis* samples all strongly correlate with δ-3-carene; *P. lambertiana* and *P. flexilis* essential oil samples correlate strongly with β-pinene; and *P. monticola* samples positively correlate with β-pinene, α-pinene, and δ-3-carene. Unless cones are present, it is generally difficult to distinguish *P. flexilis* from P*. albicaulis* [6]. However, the leaf volatile compositions readily distinguish the *P. albicaulis* from the other members of the *Strobus* group. *Pinus albicaulis* essential oils are dominated by δ-3-carene, while the other *Pinus* essential oils are rich in α- and β-pinenes. Thus, based on essential oil compositions, it may be possible to more confidently identify members of the *Strobus* subgenus.

### 2.5. Pinus sabiniana Douglas

Leaves of *P. sabiniana* were collected from three individual trees growing near Paradise, California. Hydrodistillation gave colorless essential oils in yields of 2.33 to 2.45%. The gas chromatographic analysis of the leaf essential oils resulted in the identification of 96 components, accounting for 99.6%, 99.6%, and 99.8% of the total compositions (Table 6). The leaf essential oils showed notable variation in compositions, depending on the elevation of the collection site, whether in Butte Creek Canyon (samples #1 and #2) or on the top of the butte in Paradise (sample #3). Thus, for example, the major component in samples #1 and #2 was α-pinene (65.0% and 61.2%) but only 15.8% in sample #3, while the major component in sample #3 was limonene (54.9%) but only 1.5% and 1.4% in samples #1 and #2. Other major components in the leaf essential oils were (Z)-β-ocimene (7.9%, 11.3%, and 9.6%), β-pinene (6.6%, 6.6%, and 2.0%), and myrcene (3.8%, 4.9%, and 5.7%). The leaf essential oil compositions in this study are in qualitative agreement with a previous study on *P. sabiniana* from Placerville, California, that showed α-pinene (39.1%), limonene (10.5%), β-phellandrene (10.4%), thunbergol (4.7%), (*Z*)-β-ocimene (4.6%), methyl chavicol (4.5%), myrcene (3.6%), and β-pinene (3.3%) to be the major components [23]. Note that in addition to elevation, a recent wildfire episode (the so-called Camp Fire, 8 November 2018 [29]) may also have affected the trees in this study.

### 2.6. Enantiomeric Distributions

The leaf essential oils of *P. albicaulis*, *P. flexilis*, *P. lambertiana*, *P. monticola*, and *P. sabiniana* were analyzed by enantioselective gas chromatography in order to determine the enantiomeric distributions of chiral monoterpenoid components. The enantiomeric distributions of chiral monoterpenoid components found in the *Pinus* species are compiled in Table 7, Table 8, Table 9, Table 10 and Table 11, respectively.

Only one enantiomer of α-thujene was observed in *P. albicaulis* or *P. flexilis*. Unfortunately, the RI values for (+)- and (−)-α-thujene are very similar, so the assignment of (−)-α-thujene is tentative. Interestingly, the enantiomeric distribution for α-pinene showed (+)-α-pinene to be dominant in *P. flexilis* from southern Utah, whereas (−)-α-pinene dominated *P. albicaulis*. It would be tempting to suggest that the enantiomeric distribution of α-pinene could serve to differentiate *P. albicaulis* from *P. flexilis*, but (−)-α-pinene dominated *P. flexilis* from southern Idaho [14]. Indeed, the enantiomeric distribution of α-pinene in *Pinus* species is variable both between species and within species [14,30]. Nevertheless, (−)-α-pinene was dominant in *P. lambertiana*, *P. monticola*, and *P. sabiniana*.

(−)-Limonene often dominates the essential oils of *Pinus* species [14], but there are exceptions (e.g., *Pinus mugo* Turra [31], *Pinus sylvestris* L. [31], and *Pinus uncinata* subsp. *uliginosa* (G.E.Neumann ex Wimm.) Businský [32]). In this work, (−)-limonene was dominant in *P. albicaulis*, *P. flexilis*, and *P. lambertiana*, but the limonene distribution was variable in *P. monticola* and *P. sabiniana*.

Similarly, (−)-β-phellandrene was dominant in *P. albicaulis*, *P. flexilis*, *P. lambertiana*, and *P. sabiniana*, consistent with observations in *Pinus ponderosa* Douglas ex C. Lawson var. *ponderosa*, *Pinus contorta* Douglas ex Loudon subsp. *contorta*, *P. flexilis* from Idaho [14], and *Pinus contorta* subsp. *murrayana* (Balf.) Engelm. [33]. In contrast, however, β-phellandrene was virtually racemic in *P. monticola*. The (−)-enantiomers dominated camphene, β-pinene, terpinen-4-ol, and α-terpineol in *P. albicaulis*, *P. flexilis*, *P. lambertiana*, *P. monticola*, and *P. sabiniana*. (−)-Sabinene was dominant in the leaf essential oils of *P. albicaulis* and *P. flexilis*. Only one enantiomer was observed for δ-3-carene in *P. albicaulis*, *P. flexilis*, and *P. monticola*. The observed RI value is consistent with (+)-δ-3-carene, but a reference for (−)-δ-3-carene was not available for comparison. The only enantiomer of borneol was (−)-borneol in the essential oils of *P. monticola*, which is consistent with those observed in the essential oils of *P. contorta latifolia* [33], *P. flexilis* from Idaho [14], *Pinus edulis* Engelm., and *Pinus monophylla* Torr. & Frém. [30].

## 3. Materials and Methods

### 3.1. Collection and Identification

The collection details are summarized in Table 12. Leaves (needles) were collected from individual trees at the locations indicated. Several branch tips from each individual tree were collected. Voucher specimens were deposited with the University of Alabama in Huntsville herbarium. Identification in the field was carried out by W.N. Setzer and later verified by comparison with herbarium samples from the C.V. Starr Virtual Herbarium, New York Botanical Garden (https://sweetgum.nybg.org/science/vh/, accessed on 12 December 2024). The leaves were stored frozen (−20 °C) until hydrodistillation.

### 3.2. Essential Oils

The leaf essential oils of the *Pinus* species were obtained by hydrodistillation of each tree sample using a Likens–Nickerson apparatus for four hours with continuous extraction of the distillate with dichloromethane to give colorless essential oils. The hydrodistillation yields are summarized in Table 12.

### 3.3. Gas Chromatographic Analyses

The Pinus leaf essential oils were subjected to gas chromatographic analyses (GC-FID, GC-MS, and enantioselective GC-MS) as previously described [14,33]. One replicate was carried out for each essential oil.

### 3.4. Statistical Analyses

The agglomerative hierarchical cluster analyses (HCA) and principal component analyses (PCA) were carried out using XLSTAT v. 2018.1.1.62926 (Addinsoft, Paris, France). In the case of HCA on *P. albicaulis*, the percentages of the major components (δ-3-carene, α-pinene, β-pinene, limonene, myrcene, β-phellandrene, α-terpinyl acetate, α-cadinol, terpinolene, camphene, α-terpineol, and germacrene D) were used, Pearson correlation was used to measure similarity, and the unweighted pair group method with arithmetic average (UPGMA) was used for cluster definition. For the HCA on the *Strobus* group, the concentrations of the most abundant components (β-pinene, α-pinene, δ-3-carene, limonene, α-terpineol, β-phellandrene, myrcene, α-cadinol, germacrene D, (*E*)-β-caryophyllene, terpinolene, camphene, α-terpinyl acetate, and *trans*-β-elemene) were used. Dissimilarity was used to determine clusters considering Euclidean distance, and Ward’s method was used to define agglomeration. A PCA, type Pearson correlation, was carried out to verify the results of the HCA using the same major components. Student’s *t*-test [34] was used to compare the major components in the *P. albicaulis* samples using Minitab^®^ 18 (Minitab Inc., State College, PA, USA). Differences of *p* < 0.05 were considered to be statistically significant.

## 4. Conclusions

This report, for the first time, presents the leaf essential oil compositions, including enantiomeric distributions, for *P. albicaulis* and *P. lambertiana*. In addition, the enantiomeric distributions have been determined for *P. monticola* and *P. sabiniana*. The pine essential oils examined in this study reveal high concentrations of α-pinene and β-pinene, typical for pine species. There are, however, interspecific variations in compounds such as δ-3-carene, (*E*)-β-caryophyllene, and germacrene D. The enantiomeric distributions are, in general, inconsistent throughout the genus. However, the differences observed may be useful in identifying species, hybrids, or essential oil adulteration. An obvious limitation of this study is that samples were obtained opportunistically, with only a few samples of each species obtained from limited geographical locations. While this work does provide additional insight into the essential oil compositions of several understudied *Pinus* species in western North America, additional research is needed to confirm these observations. For example, are the leaf essential oil compositions of *P. albicaulis* and *P. flexilis* relatively consistent throughout their range? Additional collections and analyses of the *Pinus* species from other locations in their respective ranges would provide additional information regarding the volatile phytochemistry of these pine trees. Depending on availability (e.g., *Pinus albicaulis* is a threatened species), the essential oils may be useful in pharmaceuticals, cosmetics, or aromatherapy.

## Figures and Tables

**Table 1:** Leaf essential oil compositions (percentages) of *Pinus albicaulis* Engelm. from Wyoming and from California.

RI_calc_	RI_db_	Compounds	*P. alb.* WY#1	*P. alb.* WY#2	*P. alb.* CA#1	*P. alb.* CA#2
779	773	Prenol	tr	0.1	tr	tr
788	797	(3*Z*)-Hexenal	tr	0.1	tr	tr
798	801	Hexanal	0.2	0.3	0.1	0.2
850	849	(2*E*)-Hexenal	1.1	1.6	0.6	0.7
851	847	(3*E*)-Hexenol	0.5	1.3	1.9	1.7
861	864	(2*E*)-Hexenol	tr	0.1	tr	tr
864	867	1-Hexanol	0.1	0.2	0.1	0.1
879	880	Santene	0.2	0.1	0.1	0.1
922	923	Tricyclene	0.3	0.2	0.1	0.2
925	925	α-Thujene	1.1	0.6	0.5	0.5
933	933	α-Pinene	12.4	12.6	8.9	7.8
947	948	α-Fenchene	tr	tr	tr	tr
949	950	Camphene	3.8	1.9	1.4	1.5
968	970	3,7,7-Trimethylcyclohepta-1,3,5-triene	0.1	0.1	tr	tr
971	971	Sabinene	1.3	0.8	0.5	0.8
977	978	β-Pinene	2.8	5.2	3.3	2.4
988	989	Myrcene	3.0	4.2	7.1	6.7
1005	1005	(3*Z*)-Hexenyl acetate	-	-	0.1	0.2
1007	1007	α-Phellandrene	0.1	0.7	0.7	0.4
1009	1009	δ-3-Carene	37.3	32.9	22.0	28.3
1017	1018	α-Terpinene	0.5	0.4	0.4	0.5
1019	1022	*m*-Cymene	tr	tr	tr	tr
1025	1025	*p*-Cymene	0.2	0.4	0.4	0.3
1029	1030	Limonene	8.7	9.7	8.4	6.8
1031	1031	β-Phellandrene	2.0	11.3	2.9	2.0
1032	1032	1,8-Cineole	tr	tr	-	-
1035	1035	(*Z*)-β-Ocimene	tr	tr	tr	tr
1037	1041	2-Heptyl acetate	0.3	0.3	0.3	0.5
1045	1045	(*E*)-β-Ocimene	0.1	0.2	0.3	0.2
1057	1057	γ-Terpinene	0.9	0.7	0.6	0.8
1084	1086	Terpinolene	5.1	3.6	3.3	4.1
1090	1090	2-Nonanone	-	-	0.1	0.1
1091	1091	*p*-Cymenene	0.1	0.1	-	-
1099	1101	Linalool	tr	tr	tr	tr
1106	1107	Nonanal	tr	tr	tr	tr
1125	1124	*cis-p*-Menth-2-en-1-ol	0.2	0.3	0.2	0.1
1128	1127	α-Campholenal	0.1	0.1	tr	0.1
1143	1142	*trans-p*-Menth-2-en-1-ol	0.1	0.2	0.1	0.1
1155	1156	Camphene hydrate	0.1	0.1	tr	tr
1172	1171	*p*-Mentha-1,5-dien-8-ol	0.1	0.1	tr	tr
1181	1180	Terpinen-4-ol	1.7	1.5	1.6	1.7
1186	1187	Cryptone	-	0.1	-	-
1188	1188	*p*-Cymen-8-ol	0.1	0.2	0.1	0.1
1196	1195	α-Terpineol	0.5	0.7	1.2	0.6
1198	1198	Estragole (=Methyl chavicol)	tr	0.3	1.6	0.7
1229	1229	Thymyl methyl ether	0.1	0.2	-	-
1233	1233	2-Nonyl acetate	0.1	-	-	-
1272	1274	Cyclooctyl acetate	0.1	-	0.1	0.2
1283	1285	Bornyl acetate	3.4	1.4	1.8	1.6
1291	1293	2-Undecanone	tr	-	0.1	0.1
1332	1332	4-Terpinyl acetate	0.3	tr	0.3	0.3
1334	1335	δ-Elemene	tr	tr	-	tr
1342	1346	α-Terpinyl acetate	1.2	0.2	14.4	9.4
1346	1346	α-Cubebene	-	0.1	-	-
1357	1357	2-Methylundecanal	tr	tr	-	-
1358	1361	Neryl acetate	tr	tr	-	-
1374	1375	α-Copaene	tr	0.1	0.1	tr
1378	1378	Geranyl acetate	0.1	0.1	0.1	0.2
1381	1383	*cis*-β-Elemene	tr	-	tr	tr
1382	1382	β-Bourbonene	tr	0.1	tr	tr
1386	1387	β-Cubebene	-	-	tr	tr
1388	1390	*trans*-β-Elemene	0.3	tr	0.3	0.4
1417	1417	(*E*)-β-Caryophyllene	tr	0.3	0.7	0.2
1428	1430	β-Copaene	tr	tr	tr	tr
1432	1432	*trans*-α-Bergamotene	-	tr	-	-
1439	1440	(*Z*)-β-Farnesene	-	0.1	-	-
1447	1446	*cis*-Muurola-3,5-diene	tr	tr	tr	tr
1451	1451	(*E*)-β-Farnesene	tr	tr	tr	tr
1454	1454	α-Humulene	-	tr	0.2	0.1
1461	1463	*cis*-Cadina-1(6),4-diene	tr	-	tr	tr
1470	1472	*trans*-Cadina-1(6),4-diene	tr	tr	0.1	0.1
1473	1475	γ-Muurolene	0.1	tr	0.1	0.2
1480	1480	Germacrene D	0.6	0.7	1.4	1.9
1488	1489	β-Selinene	0.1	-	-	-
1490	1492	*trans*-Muurola-4(14),5-diene	0.1	tr	0.1	0.1
1494	1497	Bicyclogermacrene	0.3	0.1	0.2	0.3
1496	1497	Valencene	-	-	0.1	0.1
1497	1497	α-Muurolene	0.2	0.1	0.3	0.5
1506	1508	β-Bisabolene	0.1	0.1	-	0.1
1511	1512	γ-Cadinene	0.3	0.1	0.4	0.6
1516	1518	δ-Cadinene	0.9	0.3	1.5	2.3
1520	1519	*trans*-Calamenene	-	-	0.1	tr
1521	1521	Zonarene	0.1	0.1	0.1	0.1
1531	1533	*trans*-Cadina-1,4-diene	-	-	tr	0.1
1535	1538	α-Cadinene	tr	tr	0.1	0.1
1561	1561	(*E*)-Nerolidol	0.1	0.1	-	-
1562	1560	Dodecanoic acid	tr	0.1	tr	tr
1574	1574	Germacra-1(10),5-dien-4β-ol	0.4	0.1	0.3	0.4
1576	1576	Spathulenol	0.2	0.2	0.2	0.2
1580	1587	Caryophyllene oxide	-	-	0.1	tr
1592	1593	Ethyl laurate	-	0.1	-	-
1600	1600	α-Oplopenone	0.1	-	0.2	0.2
1614	1614	1,10-di-*epi*-Cubenol	-	-	-	0.1
1626	1628	1-*epi*-Cubenol	0.1	tr	0.2	0.2
1641	1640	τ-Cadinol	0.5	0.1	0.9	1.3
1644	1644	τ-Muurolol	0.8	0.2	1.3	1.8
1646	1643	α-Muurolol (=δ-Cadinol)	0.4	0.1	0.5	0.7
1656	1655	α-Cadinol	2.2	0.5	3.4	4.4
1686	1688	α-Bisabolol	0.2	0.2	-	0.1
1764	1769	Benzyl benzoate	-	-	0.1	0.1
1814	1817	Hexadecanal	tr	tr	0.1	tr
1988	1989	Manoyl oxide	0.3	0.3	0.4	0.3
2037	2038	Thunbergol A	0.2	0.2	tr	0.2
2053	2053	Manool	0.5	0.1	0.1	-
2217	---	*iso*-Pimarinal ^a^	0.1	0.1	0.1	0.1
2225	2245	Palustral	0.2	0.2	0.1	0.1
		Monoterpene hydrocarbons	79.9	85.6	60.8	63.3
		Oxygenated monoterpenoids	8.0	5.1	19.8	14.3
		Sesquiterpene hydrocarbons	3.0	2.1	5.7	7.2
		Oxygenated sesquiterpenoids	5.0	1.4	7.1	9.2
		Diterpenoids	1.2	0.9	0.7	0.6
		Benzenoid aromatics	tr	0.3	1.6	0.8
		Others	2.5	4.2	3.5	3.8
		Total identified	99.5	99.6	99.2	99.3

RI_calc_ = The calculated retention index determined with respect to a homologous series of *n*-alkanes on a ZB-5ms column [24]. RI_db_ = Retention index values obtained from the databases [25,26,27,28]. ^a^ A reference RI value was not available, but the MS showed a 94% match. WY = Wyoming. CA = California. Percentages were determined based on peak areas.

**Table 2:** Comparison (*t*-test) of concentrations of major components of *Pinus albicaulis* leaf essential oils collected from Wyoming and California.

Compounds	Average Concentration ± Standard Deviation (%)		*p*-Value
	Wyoming	California	
δ-3-Carene	35.10 ± 3.11	25.15 ± 4.45	0.235
α-Pinene	12.50 ± 0.14	8.35 ± 0.78	0.085
β-Pinene	4.00 ± 1.70	2.85 ± 0.64	0.534
Limonene	9.20 ± 0.71	7.60 ± 1.13	0.339
Myrcene	3.60 ± 0.85	6.90 ± 0.28	0.121
β-Phellandrene	6.65 ± 6.58	2.45 ± 0.64	0.534
α-Terpinyl acetate	0.70 ± 0.71	11.90 ± 3.54	0.142
Terpinolene	4.35 ± 1.06	3.70 ± 0.57	0.584

**Table 3:** Leaf essential oil compositions (percentages) of *Pinus flexilis* E. James collected in southern Utah.

RI_calc_	RI_db_	Compounds	*P.flex.* UT#1	*P.flex.* UT#2	*P.flex.* UT#3
779	773	Prenol	0.2	0.1	tr
788	797	(3*Z*)-Hexenal	-	-	0.1
798	801	Hexanal	0.1	0.1	0.1
850	849	(2*E*)-Hexenal	-	-	0.8
851	847	(3*E*)-Hexenol	2.4	1.1	0.5
879	880	Santene	tr	tr	-
922	923	Tricyclene	0.2	0.4	0.1
925	925	α-Thujene	0.4	0.5	0.1
933	933	α-Pinene	22.9	37.5	19.8
947	948	α-Fenchene	tr	tr	tr
949	950	Camphene	2.0	4.8	0.5
952	953	Thuja-2,4(10)-diene	tr	tr	tr
971	971	Sabinene	2.4	0.1	0.1
977	978	β-Pinene	11.1	34.9	54.8
988	989	Myrcene	4.0	2.0	2.9
1005	1005	(3*Z*)-Hexenyl acetate	0.5	0.1	0.1
1007	1007	α-Phellandrene	0.1	0.1	0.1
1009	1009	δ-3-Carene	0.1	0.9	0.7
1012	1012	Hexyl acetate	tr	-	tr
1017	1018	α-Terpinene	0.6	0.1	0.1
1025	1025	*p*-Cymene	0.2	0.1	tr
1029	1030	Limonene	7.4	2.7	2.7
1031	1031	β-Phellandrene	5.1	1.8	2.3
1035	1035	(*Z*)-β-Ocimene	tr	tr	tr
1045	1045	(*E*)-β-Ocimene	0.1	tr	tr
1057	1057	γ-Terpinene	1.0	0.2	0.1
1084	1086	Terpinolene	2.4	1.3	0.8
1091	1091	*p*-Cymenene	0.1	-	tr
1097	1099	6-Camphenone	tr	tr	-
1099	1101	Linalool	0.4	0.1	0.1
1100	1100	Undecane	-	-	tr
1106	1107	Nonanal	0.1	tr	tr
1120	1119	*endo*-Fenchol	-	0.1	-
1125	1124	*cis-p*-Menth-2-en-1-ol	0.2	tr	tr
1128	1127	α-Campholenal	0.1	0.1	0.1
1141	1141	*trans*-Pinocarveol	-	0.1	-
1143	1142	*trans-p*-Menth-2-en-1-ol	0.1	tr	0.1
1155	1156	Camphene hydrate	0.2	0.2	0.1
1163	1164	Pinocarvone	-	tr	tr
1172	1171	*p*-Mentha-1,5-dien-8-ol	tr	0.1	tr
1181	1180	Terpinen-4-ol	2.5	0.3	0.3
1188	1188	*p*-Cymen-8-ol	0.1	-	-
1196	1195	α-Terpineol	1.5	2.6	4.9
1208	1208	Verbenone	-	0.1	tr
1229	1229	Thymyl methyl ether	0.1	0.1	0.1
1252	1252	Chavicol	1.0	0.7	0.2
1255	1257	6-Undecanone	tr	tr	0.2
1272	1274	Cyclooctyl acetate	-	-	0.1
1283	1285	Bornyl acetate	0.1	0.2	0.2
1291	1293	2-Undecanone	0.7	0.5	0.5
1357	1357	2-Methylundecanal	0.2	0.2	0.1
1374	1375	α-Copaene	0.1	tr	tr
1378	1378	Geranyl acetate	0.1	-	0.1
1382	1382	β-Bourbonene	0.4	tr	tr
1388	1390	*trans*-β-Elemene	0.1	-	tr
1394	1393	2-Dodecanone	0.1	tr	tr
1410	1410	Dodecanal	0.1	-	-
1428	1430	β-Copaene	0.1	tr	-
1447	1446	*cis*-Muurola-3,5-diene	0.1	-	-
1461	1463	*cis*-Cadina-1(6),4-diene	0.1	-	tr
1470	1472	*trans*-Cadina-1(6),4-diene	0.1	tr	tr
1473	1475	γ-Muurolene	0.4	0.1	0.1
1480	1480	Germacrene D	4.6	0.6	0.8
1490	1492	*trans*-Muurola-4(14),5-diene	0.2	tr	tr
1494	1497	Bicyclogermacrene	0.6	0.1	tr
1494	1494	2-Tridecanone	-	0.1	0.1
1497	1497	α-Muurolene	0.5	0.1	0.1
1511	1512	γ-Cadinene	0.7	0.1	0.1
1516	1518	δ-Cadinene	2.5	0.5	0.5
1521	1521	Zonarene	0.1	tr	tr
1535	1538	α-Cadinene	0.1	tr	tr
1562	1560	Dodecanoic acid	0.3	-	0.4
1576	1576	Spathulenol	1.8	0.3	0.4
1592	1593	Ethyl laurate	0.2	-	-
1614	1614	1,10-di-*epi*-Cubenol	0.1	tr	-
1625	1624	Muurola-4,10(14)-dien-1β-ol	0.1	-	-
1626	1628	1-*epi*-Cubenol	0.4	0.1	0.1
1641	1640	τ-Cadinol	2.3	0.6	0.4
1644	1644	τ-Muurolol	2.8	0.7	1.1
1646	1643	α-Muurolol (=δ-Cadinol)	1.2	0.3	-
1656	1655	α-Cadinol	8.3	2.0	1.9
1814	1817	Hexadecanal	0.2	-	-
1916	1923	Cembrene	-	tr	0.1
1988	1989	Manoyl oxide	0.2	0.1	-
2037	2038	Thunbergol A	0.2	-	0.2
2225	2245	Palustral	0.4	0.1	0.1
		Monoterpene hydrocarbons	60.1	87.4	85.0
		Oxygenated monoterpenoids	5.2	3.9	5.9
		Sesquiterpene hydrocarbons	10.7	1.6	1.5
		Oxygenated sesquiterpenoids	16.9	4.0	3.9
		Diterpenoids	0.7	0.2	0.3
		Benzenoid aromatics	1.0	0.7	0.2
		Others	4.9	2.1	3.0
		Total identified	99.6	99.9	99.9

RI_calc_ = The calculated retention index determined with respect to a homologous series of *n*-alkanes on a ZB-5ms column [24]. RI_db_ = Retention index values obtained from the databases [25,26,27,28]. UT = Utah. Percentages were determined based on peak areas.

**Table 4:** Leaf essential oil compositions (percentages) of *Pinus lambertiana* from northern California.

RI_calc_	RI_db_	Compounds	*P.lamb.* #1	*P.lamb.* #2	*P.lamb.* #3	*P.lamb.* #4
800	801	Hexanal	0.1	tr	tr	tr
830	831	Furfural	tr	0.1	tr	tr
849	849	(2*E*)-Hexenal	0.3	0.2	0.2	0.1
852	853	(3*Z*)-Hexenol	-	-	-	0.1
857	---	3-Methyl-3-butenoic acid	0.1	0.4	0.1	tr
879	878	3-Methyl-2-butenoid acid (=Crotonic acid)	0.1	0.3	0.1	0.1
923	923	Tricyclene	0.1	0.1	0.2	tr
925	925	α-Thujene	tr	tr	tr	tr
933	933	α-Pinene	13.2	14.5	20.3	11.4
947	948	α-Fenchene	tr	tr	0.1	tr
949	950	Camphene	1.2	1.4	1.6	0.3
953	953	Thuja-2,4(10)-diene	tr	tr	tr	tr
972	972	Sabinene	0.2	0.1	0.1	0.1
978	978	β-Pinene	41.5	36.7	46.4	28.9
989	989	Myrcene	1.7	1.8	1.8	1.0
1005	1004	*p*-Mentha-1(7),8-diene	tr	tr	tr	tr
1007	1007	α-Phellandrene	0.1	0.1	0.1	0.1
1009	1009	δ-3-Carene	0.1	0.5	0.1	0.2
1015	1015	1,4-Cineole	tr	tr	0.1	tr
1017	1017	α-Terpinene	0.1	0.1	0.2	0.1
1025	1025	*p*-Cymene	tr	tr	0.1	tr
1029	1030	Limonene	2.5	2.7	3.2	1.6
1031	1031	β-Phellandrene	3.1	1.6	2.3	2.3
1035	1034	(*Z*)-β-Ocimene	tr	tr	tr	tr
1044	1045	Phenylacetaldehyde	tr	tr	tr	tr
1046	1046	(*E*)-β-Ocimene	0.2	tr	tr	tr
1058	1058	γ-Terpinene	0.1	0.1	0.3	0.1
1070	1069	*cis*-Linalool oxide (furanoid)	-	-	tr	tr
1072	1072	Pinol	tr	tr	0.1	0.1
1085	1086	Terpinolene	1.0	1.3	1.4	0.6
1090	1091	*p*-Cymenene	-	0.1	0.1	tr
1100	1101	Linalool	-	-	0.2	0.1
1100	1100	Undecane	0.2	0.2	-	0.1
1105	1104	Nonanal	tr	tr	tr	tr
1119	1120	*endo*-Fenchol	tr	-	tr	-
1125	1124	*cis-p*-Menth-2-en-1-ol	tr	tr	tr	tr
1127	1127	α-Campholenal	0.1	0.3	0.1	tr
1130	1131	Terpin-3-en-1-ol	tr	tr	tr	tr
1138	1137	Nopinone	tr	tr	tr	tr
1156	1156	Camphene hydrate	0.1	0.1	0.1	0.1
1160	1160	*trans*-Pinocamphone	tr	tr	0.1	tr
1163	1164	Pinocarvone	tr	tr	tr	tr
1172	1171	*p*-Mentha-1,5-dien-8-ol	tr	tr	0.1	0.1
1177	1176	*cis*-Pinocamphone	tr	0.1	tr	0.1
1181	1180	Terpinen-4-ol	0.2	0.1	0.4	0.1
1188	1188	*p*-Cymen-8-ol	tr	tr	tr	tr
1196	1195	α-Terpineol	5.1	4.2	6.9	2.6
1198	1198	Methyl chavicol (=Estragole)	tr	-	1.1	0.3
1207	1206	Decanal	tr	tr	tr	tr
1229	1229	Thymyl methyl ether	tr	tr	-	-
1252	1252	Chavicol	-	0.1	0.1	-
1292	1293	2-Undecanone	tr	tr	-	-
1300	1300	Tridecane	0.2	0.1	-	0.1
1332	1330	Bicycloelemene	-	-	tr	tr
1335	1335	δ-Elemene	-	-	0.2	0.1
1347	1348	α-Cubebene	tr	tr	tr	tr
1350	1348	α-Longipinene	tr	-	-	tr
1369	1370	α-Ylangene	tr	tr	tr	tr
1376	1375	α-Copaene	0.1	0.1	tr	0.2
1384	1385	β-Bourbonene	0.1	0.3	0.1	0.1
1387	1385	α-Bourbonene	-	tr	tr	-
1388	1387	β-Cubebene	tr	tr	tr	tr
1389	1390	*trans*-β-Elemene	0.1	0.3	0.1	0.3
1409	1411	Longifolene	0.1	tr	0.1	0.4
1410	1410	Dodecanal	0.2	0.1	0.1	0.2
1419	1417	(*E*)-β Caryophyllene	10.0	10.1	2.5	16.6
1430	1430	β-Copaene	0.1	0.2	0.1	0.1
1440	1440	(*Z*)-β-Farnesene	-	0.1	-	0.1
1441	1442	Guaia-6,9-diene	-	-	tr	tr
1449	1450	*trans*-Muurola-3,5-diene	tr	tr	-	-
1452	1452	(*E*)-β-Farnesene	tr	0.1	0.1	0.1
1455	1454	α-Humulene	1.7	1.7	0.4	2.9
1462	1463	*cis*-Muurola-4(14),5-diene	tr	tr	-	0.1
1468	1467	9-epi-(*E*)-Caryophyllene	tr	0.1	tr	0.1
1472	1472	*cis*-Cadina-1(6),4-diene	0.1	tr	tr	0.1
1475	1475	γ-Muurolene	0.3	0.4	0.2	0.5
1481	1480	Germacrene D	4.4	13.0	4.8	9.4
1489	1490	Prenyl benzoate	0.1	0.1	tr	-
1489	1490	Aristolochene	-	-	-	0.1
1491	1492	*trans*-Muurola-4(14),5-diene	0.1	0.1	tr	0.1
1495	1497	Bicyclogermacrene	tr	-	-	0.1
1495	1495	2-Tridecanone	-	tr	tr	-
1496	1501	*epi*-Zonarene	0.1	-	-	-
1496	1497	α-Selinene	-	tr	tr	0.1
1498	1500	α-Muurolene	0.6	0.3	0.1	0.9
1502	1503	β-Himachalene	tr	0.1	tr	tr
1503	1504	(*E*,*E*)-α-Farnesene	tr	0.1	0.1	0.1
1510	1511	(*Z*)-γ-Bisabolene	-	0.1	-	0.1
1514	1514	γ-Cadinene	0.8	0.3	0.1	1.0
1518	1518	δ-Cadinene	2.9	1.0	0.3	3.9
1523	1521	Zonarene	0.1	tr	tr	0.1
1533	1533	*trans*-Cadine-1,4-diene	0.1	tr	tr	0.1
1537	1538	α-Cadinene	0.1	tr	tr	0.2
1541	1541	(*E*)-α-Bisabolene	-	tr	0.5	0.2
1561	1560	Dodecanoic acid	0.1	0.2	tr	0.1
1562	1561	(*E*)-Nerolidol	0.2	0.3	0.6	0.2
1578	1574	Germacra-1(10),5-dien-4β-ol	0.4	0.1	tr	0.7
1583	1587	Caryophyllene oxide	0.2	0.2	0.1	0.4
1610	1611	Humulene epoxide II	tr	tr	tr	tr
1616	1616	1,10-di-*epi*-Cubenol	-	-	-	0.1
1628	1628	1-*epi*-Cubenol	0.1	tr	tr	0.1
1632	1629	*iso*-Spathulenol	-	-	0.1	-
1643	1643	τ-Cadinol	0.7	0.2	0.1	0.9
1645	1645	τ-Muurolol	1.0	0.2	0.1	1.4
1648	1651	α-Muurolol (=δ-Cadinol)	0.5	0.2	0.1	0.6
1657	1655	α-Cadinol	3.0	0.8	0.3	3.9
1817	1817	Hexadecanal	0.1	0.2	0.1	0.1
1832	1832	(2*E*,6*E*)-Farnesyl acetate	-	-	0.3	tr
1922	1930	Cembrene	0.1	0.1	tr	0.1
1964	1966	Pimaradiene	-	0.4	tr	1.1
1987	1997	(*Z*)-9,17-Octadecadienal	-	0.1	tr	tr
1992	1989	Manoyl oxide	tr	0.1	0.1	0.1
1994	1995	(9*Z*)-Octadecenal	-	0.2	0.1	tr
1996	2000	9β-*iso*-Pimara-7,15-diene	-	-	0.1	0.1
2010	2012	Verticilla 4(20),7,11-triene	tr	tr	tr	0.1
2013	2007	18-Norabieta-8,11,13-triene	-	-	0.1	0.1
2053	2053	Manool	-	0.2	0.1	0.1
2084	2086	Abietadiene	tr	tr	tr	tr
2181	2180	Sandaracopimarinal	-	0.4	tr	1.1
2221	2204	*iso*-Pimarinal	-	-	0.1	0.1
2229	2243	Palustral	0.1	0.1	0.1	0.1
2233	2265	Levopimarinal	tr	tr	tr	tr
2245	2257	Methyl sandaracopimarate	-	0.2	tr	0.1
2261	2266	Dehydroabietal	tr	tr	tr	tr
2306	2312	Abietal	tr	tr	tr	tr
2312	2325	Methyl daniellate	0.2	0.2	0.1	0.1
		Monoterpene hydrocarbons	65.0	61.1	78.2	46.6
		Oxygenated monoterpenoids	5.4	4.8	8.1	3.0
		Sesquiterpene hydrocarbons	21.7	28.3	9.6	38.1
		Oxygenated sesquiterpenoids	6.1	2.0	1.6	8.1
		Diterpenoids	0.4	1.5	0.7	3.1
		Benzenoid aromatics	0.1	0.2	1.2	0.3
		Others	1.3	2.2	0.7	0.8
		Total identified	100.0	100.0	99.9	99.9

RI_calc_ = The calculated retention index determined with respect to a homologous series of *n*-alkanes on a ZB-5ms column [24]. RI_db_ = Retention index values obtained from the databases [25,26,27,28]. Percentages were determined based on peak areas.

**Table 5:** Leaf essential oil compositions (percentages) of *Pinus monticola* from Mt. St. Helens, Washington, and Priest Lake, Idaho.

RI_calc_	RI_db_	Compounds	*P. mont.* WA#1	*P. mont.*WA#2	*P. mont.* WA#3	*P. mont.*ID#1	*P. mont.*ID#2	*P. mont.*ID#3
799	797	(3*Z*)-Hexenal	0.1	0.1	0.1	tr	0.1	tr
800	801	Hexanal	0.2	0.2	0.1	0.1	0.1	0.1
848	849	(2*E*)-Hexenal	2.9	2.8	2.8	1.4	2.7	1.3
850	853	(3*Z*)-Hexenol	0.1	-	0.1	-	0.1	0.1
860	860	Tiglic acid	-	-	0.1	-	-	-
883	883	3-Methyl-2-butenoic acid	0.1	-	-	-	-	0.2
896	904	Angelic acid	-	0.4	0.5	-	-	-
923	923	Tricyclene	0.1	0.1	0.1	0.1	0.1	0.1
925	925	α-Thujene	0.1	0.1	tr	tr	tr	tr
933	932	α-Pinene	15.7	12.1	14.4	9.8	12.3	11.3
947	948	α-Fenchene	0.1	0.1	0.1	0.1	0.1	0.1
949	950	Camphene	2.0	1.3	1.4	0.9	1.1	1.2
973	971	Sabinene	0.3	0.4	0.3	0.2	0.2	0.3
978	978	β-Pinene	21.2	18.7	24.1	23.1	25.6	16.7
990	989	Myrcene	4.9	4.5	4.4	4.6	4.3	3.9
1007	1006	α-Phellandrene	2.4	0.4	1.4	0.3	0.5	0.7
1010	1008	δ-3-Carene	10.8	10.6	8.2	12.9	10.3	12.6
1017	1018	α-Terpinene	0.4	0.1	0.2	tr	0.1	0.1
1025	1025	*p*-Cymene	0.3	1.3	0.6	1.3	0.9	0.6
1031	1030	Limonene	6.5	6.1	8.2	6.3	5.2	3.7
1032	1031	β-Phellandrene	6.2	4.2	5.0	3.9	4.0	3.4
1035	1034	(*Z*)-β-Ocimene	tr	tr	tr	tr	tr	tr
1038	1041	2-Heptyl acetate	0.1	tr	tr	tr	tr	tr
1046	1045	(*E*)-β-Ocimene	tr	tr	tr	tr	tr	tr
1058	1057	γ-Terpinene	0.6	0.1	0.3	0.1	0.1	0.2
1081	1082	*p*-Mentha-2,4(8)-diene	0.1	tr	0.1	0.1	0.1	0.1
1085	1086	Terpinolene	3.5	1.4	2.6	1.3	1.6	1.6
1090	1091	*p*-Cymenene	tr	tr	tr	0.1	tr	tr
1090	1090	2-Nonanone	tr	0.1	tr	0.1	tr	tr
1099	1097	α-Pinene oxide	-	-	0.1	0.1	0.1	tr
1100	1101	Linalool	-	-	-	0.1	0.1	tr
1105	1104	Nonanal	tr	tr	tr	tr	tr	tr
1107	1105	α-Thujone	0.1	tr	-	-	-	-
1119	1120	*endo*-Fenchol	-	-	-	-	0.1	-
1124	1124	*cis-p*-Menth-2-en-1-ol	0.2	0.2	0.2	0.2	0.1	0.1
1127	1127	α-Campholenal	0.1	0.1	-	-	-	tr
1132	1132	*cis*-Limonene oxide	-	tr	-	0.1	tr	tr
1138	1139	Nopinone	-	tr	-	tr	tr	tr
1142	1142	*trans-p*-Menth-2-en-1-ol	0.2	0.4	0.2	0.4	0.3	0.1
1147	1145	Camphor	0.1	-	-	-	-	-
1155	1156	Camphene hydrate	0.1	0.2	0.2	0.4	0.2	0.1
1160	1160	*trans*-Pinocamphone	0.1	0.2	0.1	0.2	0.1	0.1
1162	1164	Pinocarvone	-	-	-	0.1	tr	tr
1177	1176	*cis*-Pinocamphone	-	-	-	-	0.1	-
1182	1180	Terpinen-4-ol	0.7	1.0	0.7	1.0	0.7	0.4
1185	1184	*p*-Methylacetophenone	-	0.1	tr	0.1	0.1	tr
1188	1185	Cryptone	-	0.4	0.1	0.4	0.1	tr
1189	1186	*p*-Cymen-8-ol	0.1	0.4	0.2	0.6	0.4	0.2
1198	1195	α-Terpineol	4.1	6.1	5.2	7.8	7.7	2.1
1276	1274	Cyclooctyl acetate	0.1	0.1	tr	0.1	0.1	tr
1285	1285	Bornyl acetate	2.9	2.6	1.4	1.7	1.1	1.6
1293	1293	2-Undecanone	0.5	0.3	0.2	0.5	0.3	0.2
1347	1346	α-Terpinyl acetate	0.1	0.1	0.1	0.1	0.1	0.1
1350	1349	Citronellyl acetate	tr	0.1	tr	0.2	0.1	0.1
1358	1361	Neryl acetate	0.1	0.1	0.1	0.2	0.1	tr
1358	1357	2-Methylundecanal	tr	-	-	-	-	tr
1363	1366	Linalyl isobutyrate	-	0.2	-	0.3	0.1	tr
1378	1378	Geranyl acetate	0.1	0.2	tr	0.3	0.4	0.1
1383	1383	*cis*-β-Elemene	0.1	0.1	tr	tr	tr	1.0
1384	1382	β-Bourbonene	-	-	-	-	-	0.1
1390	1390	*trans*-β-Elemene	1.8	1.5	1.1	0.9	0.7	15.2
1394	1393	2-Dodecanone	0.1	tr	tr	0.1	tr	tr
1418	1422	β-Ylangene	-	-	-	-	-	0.1
1419	1417	(*E*)-β-Caryophyllene	0.6	0.2	0.1	0.1	0.1	0.3
1445	1451	Prenyl benzoate	-	0.1	tr	-	-	-
1453	1452	(*E*)-β-Farnesene	tr	tr	tr	-	-	0.1
1456	1454	α-Humulene	0.1	tr	tr	-	-	0.1
1462	1458	*allo*-Aromadendrene	-	-	-	-	-	0.1
1473	1475	Selina-4,11-diene	0.1	0.2	0.1	0.1	0.1	0.7
1475	1478	γ-Muurolene	-	0.1	0.1	0.1	tr	0.1
1481	1480	Germacrene D	0.3	0.1	0.1	0.1	0.1	3.6
1489	1487	β-Selinene	0.4	0.4	0.3	0.3	0.2	2.0
1492	1492	*trans*-Muurola-4(14),5-diene	-	tr	tr	-	-	0.1
1495	1494	2-Tridecanone	0.3	0.2	0.1	0.2	0.2	0.4
1495	1497	α-Selinene	0.2	0.2	0.2	0.1	0.1	1.5
1498	1500	α-Muurolene	tr	0.2	0.1	0.2	0.2	0.3
1507	1508	β-Bisabolene	0.2	0.1	0.1	0.1	0.1	-
1507	1504	Germacrene A	-	-	-	-	-	0.5
1513	1512	γ-Cadinene	0.1	0.2	0.1	0.2	0.1	0.4
1518	1518	δ-Cadinene	0.2	0.8	0.6	0.6	0.6	1.4
1521	1519	*trans*-Calamenene	-	0.1	tr	-	-	-
1522	1521	Zonarene	-	tr	tr	-	-	-
1537	1538	α-Cadinene	0.1	-	tr	-	-	0.1
1563	1560	Dodecanoic acid (=Lauric acid)	0.2	0.3	0.3	0.2	0.1	0.3
1577	1574	Germacra-1(10),5-dien-4β-ol	-	-	-	-	-	0.1
1577	1578	Spathulenol	-	0.1	0.2	-	0.2	-
1581	1587	Caryophyllene oxide	0.1	0.1	tr	-	-	-
1593	1593	Ethyl laurate	-	-	0.1	-	-	-
1615	1614	1,10-di-*epi*-Cubenol	-	0.1	0.1	0.1	0.1	-
1627	1628	1-*epi*-Cubenol	-	0.2	0.2	0.3	0.2	-
1639	1644	*allo*-Aromadendrene epoxide	-	-	-	-	-	0.2
1643	1643	τ-Cadinol	0.3	2.0	1.4	1.6	1.8	0.6
1645	1644	τ-Muurolol	0.4	2.4	1.8	2.3	2.3	0.9
1647	1651	α-Muurolol (=δ-Cadinol)	0.2	1.0	0.9	0.9	1.2	0.3
1651	1650	Pogostol	-	0.2	-	-	-	0.4
1657	1655	α-Cadinol	0.7	7.4	5.1	6.6	6.9	2.2
1660	1660	Selin-11-en-4α-ol	1.5	1.3	1.4	1.3	1.3	2.6
1662	1661	*neo*-Intermedeol	0.3	0.4	0.2	0.3	0.2	0.2
1687	1688	α-Bisabolol	0.6	0.2	0.3	0.1	0.1	-
1758	1758	Myristic acid	-	-	0.1	-	-	-
1816	1817	Hexadecanal	0.1	-	tr	-	-	-
1958	1958	Palmitic acid	-	0.2	0.1	0.1	0.1	-
1991	1989	Manoyl oxide	0.2	0.1	0.1	0.1	0.1	0.1
2009	2012	Verticilla 4(20),7,11-triene	0.1	0.1	tr	-	-	-
2146	2143	Serratol	0.1	-	0.1	-	-	-
2180	2180	Sandaracopimarinal	0.1	0.1	0.1	-	0.1	-
2209	---	(1*R*,4a*R*,5*S*)-5-((*E*)-5-Methoxy-3-methylpent-3-en-1-yl)-1,4a-dimethyl-6-methylenedecahydro-naphthalene-1-carbaldehyde ^a^	0.1	0.2	-	-	-	-
2228	2245	Palustrinal	0.5	0.2	0.4	0.1	0.1	0.2
2260	2266	Dehydroabietal	0.1	0.3	0.1	0.2	0.1	0.1
2294	2302	Methyl levopimarate	0.2	-	-	-	-	-
2305	2312	Abietal	0.1	-	-	-	-	-
2317	---	15-Beyeren-19-yl acetate ^b^	1.1	-	-	-	-	-
2329	2324	Methyl dehydroabietate	0.1	tr	tr	-	-	0.3
		Monoterpene hydrocarbons	75.2	61.4	71.2	64.9	66.4	56.4
		Oxygenated monoterpenoids	9.0	11.8	8.5	13.8	11.9	4.9
		Sesquiterpene hydrocarbons	4.3	4.1	2.9	2.6	2.2	27.4
		Oxygenated sesquiterpenoids	4.1	15.4	11.5	13.5	14.3	7.5
		Diterpenoids	2.6	1.0	0.8	0.3	0.4	0.6
		Benzenoid aromatics	0.0	0.1	tr	0.1	0.1	tr
		Others	4.7	4.8	4.7	3.3	3.9	2.7
		Total identified	99.8	98.8	99.5	98.6	99.2	99.5

RI_calc_ = The calculated retention index determined with respect to a homologous series of *n*-alkanes on a ZB-5ms column [24]. RI_db_ = Retention index values obtained from the databases [25,26,27,28]. ^a^ A reference RI value was not available, but the MS showed an 85% match. ^b^ A reference RI value was not available, but the MS showed a 94% match. WA = Washington. ID = Idaho. Percentages were determined based on peak areas.

**Table 6:** Leaf essential oil compositions (percentages) of *Pinus sabiniana* collected in Paradise, California.

RI_calc_	RI_db_	Compounds	*P.sab.* #1	*P.sab.* #2	*P.sab.* #3
783	773	Prenol	0.1	0.1	tr
801	801	Hexanal	0.1	tr	tr
831	831	Furfural	0.1	0.1	0.1
851	850	(2*E*)-Hexenal	-	tr	0.1
851	853	(3*Z*)-Hexenol	0.1	0.1	0.1
923	923	Tricyclene	0.1	0.1	tr
925	925	α-Thujene	tr	tr	tr
933	933	α-Pinene	65.0	61.2	15.8
948	948	α-Fenchene	tr	tr	tr
949	950	Camphene	1.1	1.0	0.2
953	953	Thuja-2,4(10)-diene	0.1	tr	tr
961	960	Benzaldehyde	tr	tr	tr
972	972	Sabinene	tr	tr	tr
978	978	β-Pinene	6.6	6.6	2.0
984	984	6-Methyl-5-hepten-2-one	tr	tr	tr
989	989	Myrcene	3.8	4.9	5.7
1004	1005	Octanal	tr	0.1	0.3
1006	1006	3-Ethenyl-1,2-dimethylcyclohexa-1,4-diene	tr	tr	tr
1007	1007	α-Phellandrene	0.1	0.1	tr
1009	1009	δ-3-Carene	tr	tr	tr
1017	1018	α-Terpinene	tr	tr	tr
1026	1025	*p*-Cymene	0.1	0.1	0.1
1029	1030	Limonene	1.5	1.4	54.9
1031	1031	β-Phellandrene	1.7	1.7	0.3
1035	1035	(*Z*)-β-Ocimene	7.9	11.3	9.6
1043	1043	Phenylacetaldehyde	tr	tr	tr
1045	1045	(*E*)-β-Ocimene	0.5	0.6	0.6
1058	1058	γ-Terpinene	0.1	tr	tr
1072	1072	Pinol	tr	tr	tr
1085	1086	Terpinolene	0.5	0.4	0.2
1089	1091	*p*-Cymenene	tr	-	tr
1092	1091	Rosefuran	-	tr	-
1098	1097	α-Pinene oxide	0.1	tr	-
1100	1101	Linalool	-	0.1	0.1
1100	1100	Undecane	0.1	-	-
1104	1104	Nonanal	0.1	0.1	0.1
1118	1119	*endo*-Fenchol	tr	tr	tr
1122	1122	*trans-p*-Mentha-2,8-dien-1-ol	-	-	tr
1127	1127	α-Campholenal	0.2	0.1	tr
1128	1128	(4*E*,6*Z*)-*allo*-Ocimene	0.3	0.4	0.3
1136	1137	*cis-p*-Mentha-2,8-dien-1-ol	-	-	tr
1138	1139	Nopinone	tr	tr	-
1140	1141	*trans*-Pinocarveol	0.1	0.1	-
1146	1145	Camphor	tr	tr	-
1152	1152	Citronellal	-	-	0.1
1154	1156	Camphene hydrate	0.1	tr	-
1159	1160	*trans*-Pinocamphone	0.1	0.1	-
1162	1164	Pinocarvone	0.1	0.1	tr
1171	1171	*p*-Mentha-1,5-dien-8-ol	0.3	0.2	tr
1175	1176	*cis*-Pinocamphone	tr	0.1	-
1175	1177	(3*E*,5*Z*)-Undeca-1,3,5-triene	0.3	0.8	0.4
1180	1180	Terpinen-4-ol	0.1	0.1	tr
1185	1185	(3*E*,5*E*)-Undeca-1,3,5-triene	0.1	0.1	0.1
1186	1186	*p*-Cymen-8-ol	0.1	tr	-
1195	1195	α-Terpineol	3.1	1.6	0.4
1197	1197	Methyl chavicol (=Estragole)	0.6	0.9	3.5
1206	1206	Decanal	0.3	0.5	0.8
1207	1208	Verbenone	0.2	0.1	-
1229	1229	Thymyl methyl ether	-	tr	-
1244	1246	Carvone	-	-	0.1
1254	1254	Phenylethyl acetate	-	-	tr
1351	1356	Eugenol	-	-	tr
1389	1390	*trans*-β-Elemene	0.2	0.3	0.1
1399	1403	Methyl eugenol	-	0.1	tr
1409	1410	Dodecanal	0.6	0.5	0.5
1418	1417	(*E*)-β-Caryophyllene	0.2	0.2	0.4
1436	1437	2-Phenylethyl butanoate	0.6	-	-
1446	1447	Geranylacetone	0.1	tr	tr
1452	1452	(*E*)-β-Farnesene	0.1	0.1	tr
1454	1454	α-Humulene	-	-	0.1
1480	1480	Germacrene D	0.1	0.2	0.1
1488	1487	β-Selinene	tr	0.1	0.1
1489	1489	(*Z*,*E*)-α-Farnesene	tr	tr	tr
1495	1497	α-Selinene	0.1	0.1	0.1
1503	1503	(*E*,*E*)-α-Farnesene	0.1	0.3	0.1
1517	1518	δ-Cadinene	tr	0.1	tr
1560	1560	Dodecanoic acid	0.4	0.1	0.1
1560	1560	(*E*)-Nerolidol	-	0.1	0.3
1637	1639	Phenylethyl hexanoate	0.1	-	-
1655	1655	α-Cadinol	0.2	0.1	tr
1766	1769	Benzyl benzoate	-	tr	0.2
1816	1817	Hexadecanal	tr	tr	tr
1854	1856	Phenylethyl benzoate	0.2	0.4	tr
1993	1994	Manoyl oxide	0.3	0.4	0.5
2009	2007	18-*nor*-Abieta-8,11,13-triene	0.1	0.1	0.1
2010	---	Biformene ^a^	-	-	0.1
2146	2147	(*Z*)-Abienol	0.1	0.2	tr
2178	2180	Sandaracopimarinal	tr	-	-
2227	2245	Palustral	0.1	0.1	0.1
2232	2265	Levopimarinal	tr	0.1	tr
2246	2257	Methyl sandaracopimarate	-	-	0.1
2291	2297	Methyl isopimarate	-	-	tr
2296	2302	Methyl levopimarate	0.1	tr	0.1
2330	2350	(1*R*,4a*R*,5*S*)-5-[(*E*)-5-Hydroxy-3-methylpent-3-enyl]-1,4a-dimethyl-6-methylidene-3,4,5,7,8,8a-hexahydro-2*H*-naphthalene-1-carbaldehyde	1.0	1.2	1.0
2365	2366	Neoabietic acid	0.1	0.2	0.1
2428	2441	Methyl neoabietate	0.1	tr	0.1
		Monoterpene hydrocarbons	88.9	89.3	89.3
		Oxygenated monoterpenoids	4.5	3.0	1.1
		Sesquiterpene hydrocarbons	0.6	1.3	0.9
		Oxygenated sesquiterpenoids	0.2	0.2	0.3
		Diterpenoids	0.8	0.9	1.0
		Benzenoid aromatics	1.5	1.3	3.6
		Others	2.1	2.4	2.5
		Total identified	99.6	99.6	99.8

RI_calc_ = The calculated retention index determined with respect to a homologous series of *n*-alkanes on a ZB-5ms column [24]. RI_db_ = Retention index values obtained from the databases [25,26,27,28]. ^a^ A reference RI value was not available, but the MS showed an 86% match. Percentages were determined based on peak areas.

**Table 7:** Enantiomeric distribution of chiral monoterpenoids in *Pinus albicaulis*.

Enantiomers	RI_calc_	RI_db_	WY#1	WY#2	CA#1	CA#2
(+)-α-Thujene	n.d.	950	0.0	0.0	0.0	0.0
(−)-α-Thujene	952	951	100.0	100.0	100.0	100.0
(−)-α-Pinene	974	976	94.4	85.0	80.2	86.4
(+)-α-Pinene	980	982	5.6	15.0	19.8	13.6
(−)-Camphene	1001	998	97.9	97.3	96.0	97.0
(+)-Camphene	1006	1005	2.1	2.7	4.0	3.0
(+)-Sabinene	1022	1021	3.2	7.0	8.7	4.2
(−)-Sabinene	1030	1030	96.8	93.0	91.3	95.8
(+)-β-Pinene	1027	1027	6.3	5.9	9.7	7.9
(−)-β-Pinene	1031	1031	93.7	94.1	90.3	92.1
(+)-δ-3-Carene	1049	1052	100.0	100.0	100.0	100.0
(−)-δ-3-Carene	n.d.	n.a.	0.0	0.0	0.0	0.0
(−)-Limonene	1074	1073	96.0	93.9	85.0	87.4
(+)-Limonene	1081	1081	4.0	6.1	15.0	12.6
(−)-β-Phellandrene	1083	1083	98.5	95.5	59.6	71.3
(+)-β-Phellandrene	1086	1089	1.5	4.5	40.4	28.7
(+)-Terpinen-4-ol	1295	1297	27.4	29.6	30.5	29.1
(−)-Terpinen-4-ol	1298	1300	72.6	70.4	69.5	70.9
(−)-α-Terpineol	1349	1347	-	80.0	85.7	84.3
(+)-α-Terpineol	1358	1356	-	20.0	14.3	15.7

RI_calc_ = The calculated retention index determined with respect to a homologous series of *n*-alkanes on a B-Dex 325 chiral capillary column. RI_db_ = Retention index values obtained from our own database using available reference compounds. n.d. = Compound not detected. n.a. = Reference compound not available. Percentages were determined based on peak areas.

**Table 8:** Enantiomeric distribution of chiral monoterpenoids in *Pinus flexilis*.

Enantiomers	RI_calc_	RI_db_	*P.flex.* UT#1	*P.flex.* UT#2	*P.flex.* UT#3
(+)-α-Thujene	n.d.	950	0.0	0.0	-
(−)-α-Thujene	952	951	100.0	100.0	-
(−)-α-Pinene	974	976	27.8	31.2	25.1
(+)-α-Pinene	980	982	72.2	68.8	74.9
(−)-Camphene	1001	998	88.5	93.1	70.8
(+)-Camphene	1006	1005	11.5	6.9	29.2
(+)-Sabinene	1022	1021	1.6	-	-
(−)-Sabinene	1030	1030	98.4	-	-
(+)-β-Pinene	1027	1027	2.9	2.0	1.6
(−)-β-Pinene	1031	1031	97.1	98.0	98.4
(+)-δ-3-Carene	1049	1052	-	100.0	100.0
(−)-δ-3-Carene	n.d.	n.a.	-	0.0	0.0
(−)-Limonene	1074	1073	92.3	69.2	67.0
(+)-Limonene	1081	1081	7.7	30.8	33.0
(−)-β-Phellandrene	1083	1083	98.7	98.2	99.0
(+)-β-Phellandrene	1086	1089	1.3	1.8	1.0
(+)-Terpinen-4-ol	1295	1297	26.5	31.4	29.7
(−)-Terpinen-4-ol	1298	1300	73.5	68.6	70.3
(−)-α-Terpineol	1349	1347	67.3	85.5	93.6
(+)-α-Terpineol	1358	1356	32.7	14.5	6.4

RI_calc_ = The calculated retention index determined with respect to a homologous series of *n*-alkanes on a B-Dex 325 chiral capillary column. RI_db_ = Retention index values obtained from our own database using available reference compounds. n.d. = Compound not detected. n.a. = Reference compound not available. Percentages were determined based on peak areas.

**Table 9:** Enantiomeric distribution of chiral monoterpenoids in *Pinus lambertiana*.

Enantiomers	RI_calc_	RI_db_	*P.lamb.* #1	*P.lamb.* #2	*P.lamb.* #3	*P.lamb.* #4
(−)-α-Pinene	975	976	68.8	71.5	61.7	61.0
(+)-α-Pinene	982	982	31.2	28.5	38.3	39.0
(−)-Camphene	1002	998	93.7	93.7	90.9	81.2
(+)-Camphene	1007	1005	6.3	6.3	9.1	18.8
(+)-β-Pinene	1026	1027	1.8	1.8	1.8	1.7
(−)-β-Pinene	1027	1031	98.2	98.2	98.2	98.3
(−)-Limonene	1075	1073	70.2	71.5	62.6	66.8
(+)-Limonene	1081	1081	29.8	28.5	37.4	33.2
(−)-β-Phellandrene	1083	1083	98.9	98.1	98.5	98.7
(+)-β-Phellandrene	1088	1089	1.1	1.9	1.5	1.3
(+)-Terpinen-4-ol	1293	1297	35.2	39.9	42.3	38.1
(−)-Terpinen-4-ol	1296	1300	64.8	64.1	57.7	61.9
(−)-α-Terpineol	1345	1347	95.5	95.4	93.7	94.4
(+)-α-Terpineol	1357	1356	4.5	4.6	6.3	5.6

RI_calc_ = The calculated retention index determined with respect to a homologous series of *n*-alkanes on a B-Dex 325 chiral capillary column. RI_db_ = Retention index values obtained from our own database using available reference compounds. Percentages were determined based on peak areas.

**Table 10:** Enantiomeric distribution of chiral monoterpenoids in *Pinus monticola*.

Enantiomers	RI_calc_	RI_db_	WA#1	WA#2	WA#3	ID#1	ID#2	ID#3
(−)-α-Pinene	975	976	63.1	62.3	61.3	59.2	67.7	63.2
(+)-α-Pinene	981	982	36.9	37.7	38.7	40.8	32.3	36.8
(−)-Camphene	1002	998	94.6	92.6	91.9	90.2	92.1	94.1
(+)-Camphene	1007	1005	5.4	7.4	8.1	9.8	7.9	5.9
(+)-β-Pinene	1026	1027	4.4	4.3	3.7	3.3	3.2	3.9
(−)-β-Pinene	1027	1031	95.6	95.7	96.3	96.7	96.8	96.1
(+)-δ-3-Carene	1049	1052	100.0	100.0	100.0	100.0	100.0	100.0
(−)-δ-3-Carene	n.d.	n.a.	0.0	0.0	0.0	0.0	0.0	0.0
(−)-α-Phellandrene	n.d.	1050	0.0	0.0	0.0	0.0	0.0	0.0
(+)-α-Phellandrene	1054	1053	100.0	100.0	100.0	100.0	100.0	100.0
(−)-Limonene	1075	1073	46.7	42.0	60.5	43.5	44.5	41.2
(+)-Limonene	1081	1081	53.3	58.0	39.5	56.5	55.5	58.8
(−)-β-Phellandrene	1084	1083	47.4	42.3	48.0	44.9	52.8	51.4
(+)-β-Phellandrene	1088	1089	52.6	57.7	52.0	55.1	47.2	48.6
(+)-Terpinen-4-ol	1300	1297	45.1	40.3	40.8	40.7	40.9	39.1
(−)-Terpinen-4-ol	1303	1300	54.9	59.7	59.2	59.3	59.1	60.9
(−)-Borneol	1336	1335	100.0	100.0	100.0	100.0	100.0	100.0
(+)-Borneol	n.d.	1340	0.0	0.0	0.0	0.0	0.0	0.0
(−)-α-Terpineol	1344	1347	89.7	89.6	90.5	91.8	92.7	89.5
(+)-α-Terpineol	1354	1356	10.3	10.4	9.5	8.2	7.3	10.5

RI_calc_ = The calculated retention index determined with respect to a homologous series of *n*-alkanes on a B-Dex 325 chiral capillary column. RI_db_ = Retention index values obtained from our own database using available reference compounds. n.d. = Compound not detected. n.a. = Reference compound not available. Percentages were determined based on peak areas.

**Table 11:** Enantiomeric distribution of chiral monoterpenoids in *Pinus sabiniana*.

Enantiomers	RI_calc_	RI_db_	*P.sab.* #1	*P.sab.* #2	*P.sab.* #3
(−)-α-Pinene	975	976	90.6	93.0	67.4
(+)-α-Pinene	983	982	9.4	7.0	32.6
(−)-Camphene	1001	998	88.2	89.7	76.4
(+)-Camphene	1005	1005	11.8	10.3	23.6
(+)-β-Pinene	1026	1027	7.0	6.8	8.1
(−)-β-Pinene	1030	1031	93.0	93.2	91.9
(−)-Limonene	1073	1073	44.2	45.4	98.8
(+)-Limonene	1082	1081	55.8	54.6	1.2
(−)-β-Phellandrene	1085	1083	100.0	100.0	100.0
(+)-β-Phellandrene	n.d.	1089	0.0	0.0	0.0
(−)-α-Terpineol	1347	1347	88.3	88.2	77.1
(+)-α-Terpineol	1356	1356	11.7	11.8	22.9

RI_calc_ = The calculated retention index determined with respect to a homologous series of *n*-alkanes on a B-Dex 325 chiral capillary column. RI_db_ = Retention index values obtained from our own database using available reference compounds. n.d. = Compound not detected. Percentages were determined based on peak areas.

**Table 12:** Collection and hydrodistillation details for *Pinus* species (*P. albicaulis*, *P. flexilis*, *P. lambertiana*, *P. monticola*, and *P. sabiniana*).

Sample	Voucher	Collection Date	Collection Location	Mass Leaves (g)	Mass Essential Oil (g)	% Yield
*Pinus albicaulis* WY#1	WNS-Palb-0671	18 July 2024	44°56′59″ N, 109°37′60″ W, 2901 m asl	150.90	5.2936	3.508
*Pinus albicaulis* WY#2		18 July 2024	44°56′59″ N, 109°37′59″ W, 2909 m asl	178.16	5.2639	2.955
*Pinus albicaulis* CA#1	WNS-Palb-5431	24 October 2024	40°28′32″ N, 121°28′53″ W, 2478 m asl	122.09	4.1984	3.439
*Pinus albicaulis* CA#2		24 October 2024	40°28′32″ N, 121°28′53″ W, 2478 m asl	140.82	4.8943	3.476
*Pinus flexilis* UT#1	WNS-Pflex-8033	13 September 2023	37°36′56″ N, 112°10′16″ W, 2492 m asl	43.57	1.9658	4.512
*Pinus flexilis* UT#2		13 September 2023	37°36′56″ N, 112°10′16″ W, 2492 m asl	92.13	3.6771	3.991
*Pinus flexilis* UT#3		13 September 2023	37°36′56″ N, 112°10′16″ W, 2492 m asl	42.02	2.1104	5.022
*Pinus lambertiana* CA#1		26 August 2024	40°01′10″ N, 121°31′59″ W, 1597 m asl	148.43	3.2978	2.222
*Pinus lambertiana* CA#2		26 August 2024	40°01′12″ N, 121°31′56″ W, 1607 m asl	107.51	2.3674	2.202
*Pinus lambertiana* CA#3		26 August 2024	40°01′07″ N, 121°32′01″ W, 1598 m asl	114.76	2.3039	2.008
*Pinus lambertiana* CA#4	WNS-Plamb-5403	26 August 2024	40°06′28″ N, 121°35′03″ W, 1281 m asl	120.58	2.7295	2.264
*Pinus monticola* WA#1	WNS-Pmont-0338	30 June 2024	46°09′37″ N, 122°05′42″ W, 897 m asl	72.93	1.4218	1.950
*Pinus monticola* WA#2		30 June 2024	46°09′38″ N, 122°05′42″ W, 888 m asl	93.10	1.5131	1.625
*Pinus monticola* WA#3		30 June 2024	46°09′42″ N, 122°05′51″ W, 892 m asl	122.43	2.4882	2.032
*Pinus monticola* ID#1	WNS-Pmont-0746	19 August 2024	48°33′41″ N, 116°47′56″ W, 1086 m asl	97.01	1.2760	1.315
*Pinus monticola* ID#2		19 August 2024	48°33′37″ N, 116°48′06″ W, 1089 m asl	113.74	1.9408	1.706
*Pinus monticola* ID#3		19 August 2024	48°33′28″ N, 116°48′20″ W, 1064 m asl	120.66	2.2988	1.905
*Pinus sabiniana* #1	WNS-Psab-5438	1 September 2024	39°42′57″ N, 121°43′17″ W, 98 m asl	134.79	3.3030	2.450
*Pinus sabiniana* #2		1 September 2024	39°44′39″ N, 121°40′28″ W, 168 m asl	120.68	2.8608	2.371
*Pinus sabiniana* #3		1 September 2024	39°44′35″ N, 121°39′20″ W, 421 m asl	162.75	3.7948	2.332

## Data Availability

All data are available within this manuscript.
